# Store-Operated Calcium Entry Inhibitors: Structural Diversity, Mechanisms of Action, and Optimization Strategies

**DOI:** 10.3390/ijms27209077

**Published:** 2026-10-13

**Authors:** Anastasiia A. Zakharova, Dmitriy A. Grekhnev, Elena V. Kaznacheyeva, Vladimir A. Vigont

**Affiliations:** 1 Institute of Cytology of Russian Academy of Science, Tikhoretsky Ave. 4, 194064 St. Petersburg, Russia; dima.grehnyov@yandex.ru (D.A.G.); evkazn@incras.ru (E.V.K.); vvigand@gmail.com (V.A.V.); 2 Center of Genetic Reprogramming and Gene Therapy, Institute of Cytology of Russian Academy of Science, Tikhoretsky Ave. 4, 194064 St. Petersburg, Russia

**Keywords:** calcium, store-operated calcium entry, SOCE, Orai, STIM, SOC channel inhibitors, structure–activity relationship

## Abstract

Store-operated calcium entry (SOCE) is a ubiquitous calcium influx pathway critically involved in numerous physiological processes. Dysregulated SOCE contributes to the pathogenesis of cardiovascular disorders, cancer, and neurodegenerative diseases, making SOCE modulators promising therapeutic candidates. This review provides an overview of the major chemical classes of classical SOCE inhibitors that target Orai and STIM proteins. We focus on structure–activity relationships (SARs), mechanisms of action, strategies for enhancing inhibitory potency and selectivity, and discuss the limitations and side-effect profiles of these compounds. This comprehensive analysis identifies SOC channels as promising therapeutic targets and outlines future directions for rational drug design and the development of clinically viable SOCE modulators.

## 1. Introduction

SOCE is one of the most ubiquitous pathways of calcium influx in both non-excitable cells and neurons. Alterations in SOCE were observed in many pathologies, including cardiovascular disorders [1,2,3,4], cancer [5,6,7], and neurodegenerative diseases [8,9,10]. Uncontrolled SOCE can lead to improper regulation of gene expression, abnormal accumulation of calcium in cytosol, altered calcium-mediated signaling, and mitochondria calcium overload, which is toxic to cells, contributing to cell death and tissue damage.

Inhibiting store-operated calcium (SOC) channels is a prospective strategy for treatment of these pathologies, and some negative modulators of SOCE are currently under clinical trials [11]. However, there are many compounds with SOCE inhibitory potency that differ on chemical structure and have different patterns of protein targets that they may affect. Moreover, low selectivity in action of the number of SOC channel inhibitors and possible side effects complicate their implementation in medical practice. Thus, the detailed study of the molecular mechanism of their action on different SOC channels in various cell types and clarifying the relationships between chemical structure and functions open up perspectives for the targeted modification of the pharmacophores and rational drug design. Also, the search for the novel pharmacophores with SOCE inhibitory activity remain highly actual. In this review, we focus on the main chemical groups of pharmacophores found in the most common SOCE inhibitors and the possible mechanisms of action of these agents on target proteins. We summarized published findings in this area, which allows us to identify SOC channels as one of the most promising targets in the search for new therapeutic agents and outline future promising research directions.

## 2. Major Players in the Store-Operated Calcium Entry Pathway

The concept of SOC channels was proposed in 1986 by Putney et al. [12]. According to this concept, calcium entry into non-excitable cells is regulated by the depletion of intracellular calcium stores. In cells of predominantly hematopoietic origin, an exclusively calcium-selective current characterized by inward rectification and called *I_CRAC_* (calcium current activated by calcium release) has been recorded [13]. It is now known that SOCE in various cell types is realized not only with the participation of CRAC channels, but also with the family of calcium-permeable channels, with different biophysical and pharmacological properties [14].

SOCE is mediated by the Orai and STIM (stromal interaction molecule) protein complex. Orai channels are located in the plasma membrane and represent the pore-forming structure of the CRAC channel. They are formed by the assembly of six subunits [15,16,17,18,19].

STIM1 and STIM2 proteins have been identified as calcium concentration sensors in the lumen of endoplasmic reticulum (ER) stores [20]. The N-terminus of STIM proteins, containing the calcium-binding EF-hand domain, is located in the lumen of the ER. Decrease in the calcium concentration in the ER lumen below a threshold level leads to calcium dissociation from STIM, causing a conformational change in the sensor STIM. Further, the C-terminal domain of STIM unfolds and interacts with the Orai channel in regions where the ER membrane is closest to the plasma membrane (within 15 nm) [21]. Next, SOC channels are activated, which is accompanied by the entry of calcium ions into the cell [22].

Accumulating evidence indicates that TRP channels play a significant role in calcium regulation in both excitable and non-excitable cells. Most TRP channels permeate calcium ions directly across the plasma membrane, while other members of the superfamily provide a driving force by facilitating the entry of calcium ions from the outside into the cell through calcium-conducting channels. For example, in endothelial cells [23], platelets [24], and smooth muscle cells [25], calcium entry can be mediated by TRPC1 channels. TRPC1 channels closely interact with the Orai1 calcium channel and the STIM calcium sensor [26]. Depletion of calcium stores and the formation of STIM1/Orai complexes lead to the incorporation of TRPC into the plasma membrane and prolonged SOCE channel activity in cells [27]. TRP channels exhibit varying degrees of calcium permeability, ranging from moderately permeable to completely impermeable. Nevertheless, the contribution of TRPC channels to classical SOCE remains controversial [28]. Moreover, the diversity of TRPC modulators is sufficiently broad to warrant a separate review; therefore, here we focus on inhibitors of classical SOC channels that target Orai and STIM proteins.

## 3. Orai-Associated Small-Molecule Inhibitors

### 3.1. Pyrazole Derivatives

Pyrazole derivatives feature a five-membered heterocyclic ring comprising two adjacent nitrogen atoms and three carbon atoms. The core is an aromatic system with four π-electrons delocalized within the ring and one unshared pair of electrons delocalized with π-electrons. One nitrogen acts as a pyrrole (involved in the aromatic system) and the other as a pyridine (not involved in resonance). Substitutions at various ring positions yield diverse derivatives with modified physical and chemical properties, making them valuable in pharmaceuticals and other applications, including treatment of autoimmune diseases by modulating calcium influx and cytokine production. Beyond their therapeutic potential, these compounds are also powerful tools for studying calcium signaling in various cells.

The bis(trifluoromethyl) pyrazole class of compounds, including BTP1, BTP2, and BTP3 (Table 1), was initially identified by Abbott Laboratories, as potent suppressors of NFAT activation and cytokine production in T cells [29,30,31]. Unlike conventional NFAT inhibitors, such as FK506 and cyclosporine A, BTPs uniquely impede NFAT nuclear translocation without directly targeting calcineurin, suggesting an upstream effect on calcium signaling [29,30]. Subsequent studies demonstrated that BTPs effectively suppress SOCE in various cell types at low micromolar to nanomolar concentrations, with marked selectivity over voltage-dependent calcium entry [32]. Specifically, BTP2 (also known as YM-58483) inhibited Tg-induced calcium influx in Jurkat T cells with an IC_50_ value of 100 nM, without affecting PLCγ1 phosphorylation or T-cell receptor (TCR)-mediated calcium release from intracellular stores [33]. Further studies, however, reported that BTP2 inhibits CRAC channels in human T cells with an IC_50_ of approximately 10 nM [34]. This discrepancy likely stems from differences in experimental design: the former study pre-incubated cells with BTP2 for an extended time (18–24 h) to achieve full inhibition [34], whereas the latter measured cytosolic calcium concentration only a few minutes after treatment [33]. Moreover, the inhibitory effect of BTP2 on CRAC channels is modulated by extracellular calcium concentration, with higher levels diminishing its efficacy [34].

GlaxoSmithKline’s newer pyrazole derivatives, GSK-5498A, GSK-5503A, and GSK-7975A (Table 1), have emerged as selective CRAC channel inhibitors [35,36,37]. Electrophysiological studies revealed that GSK-5498A potently suppress *I_CRAC_* with an IC_50_ of approximately 1 μM in HEK293 cells stably expressing STIM1 and Orai1 [36]. GSK-5503A and GSK-7975A inhibit STIM1-mediated Orai1 and Orai3 currents with an IC_50_ of approximately 4 μM in HEK293 cells [37].

Despite their relatively high inhibitory potency, pyrazole derivatives have notable limitations. In particular, some pyrazole-based CRAC inhibitors can affect TRP channels, complicating interpretation of their cellular effects [38,39]. Moreover, their action may extend beyond direct CRAC channel inhibition, for instance, through suppression of cytokine production, and the detailed mechanisms of action of many pyrazole derivatives remain incompletely understood. Thus, key structural determinants are still being elucidated.

SAR studies indicate that for most active pyrazoles (e.g., BTP2 and GSK-7975A), the pyrazole ring supports two aryl substituents, conferring a rigid, planar structure and significant hydrophobicity that are essential for targeting proteins involved in SOCE. Altering these substituents typically reduces or abolishes their activity [40,41]. Replacing the arylamide moiety of BTP2 with a triazole ring also modulates its inhibition potency. Riva et al. [40] reported that analogs 1S ((3-(4-hydroxyphenyl)-1H-1,2,3-triazol-1-yl)-5-(trifluoromethyl)-1H-pyrazole-4-carboxylic acid) and 3T (3-(1H-1,2,3-triazol-1-yl)-5-(trifluoromethyl)-1H-pyrazole-4-carboxylic acid), in which the arylamide is replaced by a hydroxyphenyl triazole, were approximately 8- to 33-fold (IC_50_ 0.3 ± 0.1 μM in HEK293 cells and 1.2 ± 0.2 μM in Jurkat cells) and 12-fold (IC_50_ 0.8 ± 0.1 μM in Jurkat cells) more potent than Pyr3, respectively [40].

A trifluoromethyl (CF_3_) group at the C3 position of the pyrazole ring is a hallmark of BTP2 and its analogs (e.g., Pyr2 and Pyr3), providing essential electron-withdrawing, steric, and lipophilic properties for target binding [42]. Furthermore, substitution of CF_3_ with other groups (e.g., methyl) diminishes or abolishes activity, and the CF_3_ group is thought to participate in hydrogen bonding or hydrophobic interactions at the binding site. Notably, a CF_3_ group at C3 position confers greater activity than at C5 position [38]. In contrast, pyrtriazoles and GSK-7975A bear a CF_3_ group on the benzyl substituent [41]; while this influences activity, it is not absolutely required, unlike in BTP2.

In BTP2, the most critical aryl substituent is the phenyl ring linked to the pyrazole core via nitrogen. This structural fragment is additionally substituted with another heterocyclic ring (thiadiazole), which is important for inhibiting mast cell activation. Modification of this ring may result in reduced activity [42].

Pyrazole derivatives interact with Orai family proteins through multiple complex mechanisms, encompassing extracellular blockade, bidirectional modulation, and direct alterations to channel pore geometry. Integrative analyses of studies by Derler et al. [37] and Gökçe et al. [43] elucidate these interactions and identify key amino acid residues governing binding specificity. Using patch-clamp recording of *I_CRAC_* in RBL-1 cells and computational modeling, Gökçe et al. [43] found that the initial step for most pyrazoles occurs at the extracellular surface, specifically within the region of the pore-forming M1 helices from two adjacent subunits. The authors showed by molecular modeling and docking that for BTP2 and GSK-7975A, this is the only functionally significant site, where the CF_3_ groups bind to Val106 and Val198, and the nitrogen of the pyrazole ring binds to Gln109 [43]. GSK-7975A additionally interacts with Glu107 in the selectivity filter, as evidenced by a sharp decrease in sensitivity upon the E106D mutation. In the case of Pyr6, the extracellular site initiates the process, while after membrane penetrating in intracellular sites Pyr6 interacts with Arg171, Glu174, His172, and Leu175 in the M1 and M3 helices [43].

A critical functional outcome of pyrazole binding is the modification of pore geometry. Using site-directed mutagenesis Derler et al. [37] found that mutations within the selectivity filter, such as Orai1 E106D, which increases pore diameter and diminishes calcium selectivity, substantially attenuate the blocking activity of pyrazole derivatives [37]. In contrast, mutations in the outer vestibule of the pore (e.g., D110/112/114A) exert a comparatively minor effect on the blockade process relative to alterations in the selective filter itself.

### 3.2. Diphenylborate Derivatives

The 2-aminoethyl diphenylborate (2-APB; Table 1) consists of a central boron atom bonded to two phenyl groups and an oxygen atom, which is further linked to an ethyl chain terminating in a primary amine. This compound is widely used to characterize CRAC channel activity exerting a concentration-dependent effect: low concentrations (1–10 μM) potentiate CRAC currents, whereas higher concentrations (20–50 μM) inhibit them [44,45]. A remarkable feature of 2-APB is the direct activation of Orai3 channels in high doses (50 µM). This effect occurs independently of STIM1 and intracellular calcium stores [64,65]. These 2-APB-activated Orai3 channels display large outward currents, reduced calcium selectivity, enhanced permeability to monovalent cations, and an expanded pore size relative to STIM1-gated channels [45,65,66,67]. In contrast, at low doses, STIM1-mediated Orai1 and Orai3 currents are modulated in a store- and STIM1-dependent manner and remain inwardly rectifying and highly calcium selective [45,64,65].

Yamashita et al. [64] examined the gating of Orai3 in STIM1-bound and STIM1-free states and found that high doses of 2-APB functionally uncouple STIM1 and Orai3, even though FRET (Förster resonance energy transfer) measurements show no reduction in their physical interaction. The uncoupling manifests as the loss and the suppression of low-dose potentiation of Orai3 channels—both features that depend on STIM1 binding [64]. Furthermore, overexpression of STIM1 delays 2-APB-induced gating of Orai3 [64]. This effect is completely abolished by the L285D Orai3 mutant that fails to interact with STIM1, suggesting that the inhibitory effect of STIM1 can be overcome by uncoupling STIM1 from Orai3. These findings indicate that STIM1-bound channels resist 2-APB-mediated gating, while 2-APB inhibits STIM1-mediated gating, implying that the two activation states are mutually exclusive [64]. Although the mechanistic basis remains unclear, this feature could arise from increased agonist affinity of active Orai3 channels, stabilizing distinct gating modes [68].

Despite its widespread use, 2-APB has significant limitations, including bimodal effects on inositol-1,4,5-trisphosphate receptor (IP_3_)-induced calcium release, multiple, off-target actions on other cellular proteins, slow and use-dependent reversal effects, and potential toxicity. These drawbacks complicate its use as a selective inhibitor and necessitate cautious interpretation of experimental data, driving ongoing efforts to identify superior analogs.

Screening of diphenylborinate analogs by Goto et al. [69] identified DPB162-AE and DPB163-AE as ~100-fold more potent SOCE inhibitors than 2-APB with IC_50_ values of 4.8 μM, 27 nM, and 42 nM for 2-APB, DPB162-AE, and DPB163-AE, respectively (measured by calcium imaging in DT-40 cells) [69]. These compounds feature a dimeric structure with two 2-APB moieties connected by a linker. While DPB163-AE exhibited both inhibitory and potentiating effects on SOCE (similar to 2-APB) in DT-40 and HeLa cells, DPB162-AE acted purely as an inhibitor. This distinction can probably be explained by the difference in the position of the cross-bridge between the two diphenyl groups in the structurally isomeric molecules DPB162-AE and DPB163-AE. At the same time, DPB derivatives did not activate Orai3 in the absence of STIM1, possibly due to their larger molecular size hindering access to the Orai3 pore, unlike 2-APB, which is thought to act directly on the pore region independently of STIM1 [65,67,69].

SAR studies by Dellis et al. [70] further elucidated the key determinants of 2-APB activity by systematically modifying three regions: the ethanolamine chain, the boron–oxygen core, and the two phenyl rings. Diphenylborinic anhydride (DPBA), characterized by the presence of four phenyl rings and the absence of an ethanolamine group, was shown to be three- to five-fold more potent than 2-APB in inhibiting platelet SOCE (2 μM vs. 10 μM). In BL41 B cells, DPBA potentiated SOCE maximally at 3 μM and inhibited it above 10 μM, whereas in monocytic cell U937 lines and BL41, 2-APB potentiated SOCE at 10 μM and inhibited above 50 μM. These data suggest that the ethanolamine group is not critical for SOCE inhibition [70,71]. In addition, this compound was able to activate some TRP channels at concentrations > 100 μM [72,73]. Replacing the phenyl rings with butyl groups (yielding 2-aminoethyl dibutylborinate, or 2-ABB) preserved the biphasic effect on calcium release, with maximal potentiation and inhibition observed at 30 µM and above 50 µM, respectively, but with reduced efficacy compared to 2-APB and DPBA [70]. This suggests that phenyl rings are not absolutely required for SOCE modulation processes but likely facilitate it, possibly through secondary hydrophobic interactions with the channel protein, as proposed by Dobrydneva et al. [74]. Indeed, a correlation between the number of phenyl groups and the magnitude of SOCE modulation was noted: DPBA (four phenyls) > 2-APB (two) > 2-ABB (none). Dellis et al. [70] also demonstrated that the boron–oxygen bond alone does not guarantee potentiating activity.

The presence of a boron atom is also a significant factor in SOCE modulation. Replacing boron with a carbon-phosphorus core in 3-(diphenylphosphino)-1-propylamine (DP3A) abolished potentiating activity entirely, leaving only inhibitory effects at both 10 and 50 μM [70]. Additionally, halogen substitution on the phenyl rings profoundly affects SOCE modulation [75]. Schild et al. [75] systematically introduced halogens and other small substituents at each position of one of the phenyl rings and assessed activity using a fluorometric plate-reader-based calcium imaging assay (FLIPR) on the Tetra platform MDA-MB-231 breast cancer cells. The type and position of the halogen were shown to significantly influence the ability of 2-APB analogs to regulate SOCE. At 10 μM, all fluorinated analogs potentiated SOCE, as did 2-APB. Some chlorinated derivatives also potentiated depending on the substitution position, whereas bromo- and iodo-substituted analogs inhibited SOCE at this low concentration. The position of halogen was critical: *ortho*-substitution enhanced SOCE, *meta*-substitution blocked it, and *para-*substitution produced only partial or no inhibition. At the same position, the inhibitory potency of *meta*- and *para*-halogenated derivatives increased with larger atomic size and lower electronegativity (*m*-F-2-APB << *m*-Cl-2-APB < *m*-Br-2-APB < *m*-I-2-APB and *p*-F-2-APB << *p*-Cl-2-APB < *p*-Br-2-APB < *p*-I-2-APB). Moreover, effective blocking of SOCE by *meta*-substituted derivatives was also observed at 20 μM, at which 2-APB only partially inhibits [75].

The action of 2-APB on Orai family channels is a unique example of pharmacological versatility and bimodality among ion channels, capable of acting both through a direct STIM-independent effect on the Orai channel and an indirect effect through interaction with STIM [55,67,76]. Furthermore, 2-APB can exert diametrically opposed effects in both STIM-independent and STIM-dependent modulation of Orai proteins, determined by concentration, channel state, and the specifics of interaction with activator proteins. Despite the diversity of 2-APB’s mechanisms of action, this review focuses on the compound’s inhibitory effect on Orai proteins and the interaction between Orai and STIM.

Regarding the direct inhibitory effect of 2-APB, as the concentration of this compound in the membrane increases, Orai channels can transition into a state that prevents ion transport [65]. According to Sukkar et al. [77], the primary binding site for 2-APB may be transmembrane helix 1 (TM1), which forms the central pore of the Orai channel.

According to in silico modeling data of Sukkar et al. [77], after penetrating the Orai channel pore, 2-APB is anchored in the selective filter region. A crucial role in this process plays the Phe99 and Val102 residues, which together form the so-called hydrophobic hinge characterized as a structural element that ensures the conformational mobility of TM1 and is involved in the mechanism of pore opening and closing. To anchor the 2-APB molecule in the docking region, the amino group of 2-APB, positively charged at physiological pH, electrostatically binds to the negatively charged carboxyl groups of the side chains of Glu106 residues of adjacent Orai hexamer subunits. After anchoring in the selective filter, the aromatic rings of 2-APB, which make up the diphenyl portion of the molecule, are oriented toward the hinge region of TM1. Two types of hydrophobic interactions occur here. First, the aromatic ring of 2-APB engages in a *π*-*π* stacking interaction with the phenyl group of the Phe99 residue [77]. This type of interaction, characteristic of aromatic systems, provides steric and electronic conjugation between the ligand and protein, significantly enhancing binding affinity. Second, the aliphatic moieties of 2-APB form hydrophobic contacts with the side chain of the Val102 residue. Although individual hydrophobic interactions are relatively weak, their combination, coupled with electrostatic fixation in the selective filter, provides a high overall binding energy and stability of the modulator-channel complex. Ultimately, the key functional consequence of 2-APB binding is stabilization of the channel in a closed conformation.

Inhibitory effects of 2-APB are also observed within the context of STIM-dependent regulation. Lysine residue 85 (K85) within the Orai1 of the ETON region (extended transmembrane Orai1 N-terminal region, including amino acids 73–90) plays an important role in the inhibitory process [55]. Site-directed mutagenesis demonstrated that the K85E mutation (substitution of positively charged lysine with negatively charged glutamic acid) completely eliminates the inhibitory effect of 2-APB. Moreover, as demonstrated by the FRET method, K85E does not modulate the effect of 2-APB on STIM1 conformation or its binding to the channel. Instead, only the transmission of the signal from STIM1 to the pore is blocked [55]. Thus, K85 acts as a critical effector mediating the inhibitory action of 2-APB on the CRAC channel [55].

### 3.3. Polyphenols

Polyphenols constitute a diverse class of plant-derived compounds characterized by the presence of one or more benzene (aromatic) rings bearing hydroxyl (-OH) groups. Their structural complexity ranges from simple phenolic acids to highly polymerized structures and they are broadly classified into subclasses, including flavonoids, stilbenes, lignans, and phenolic acids. The specific architecture of these molecules—defined by the number of aromatic rings, degree of hydroxylation, and the nature of inter-ring linkages—determines their physical and chemical properties and biological activities, including potent antioxidant capacity. In the context of neurodegenerative diseases, polyphenols can positively influence calcium signaling by restoring calcium homeostasis, reducing ER calcium levels, and protecting from the mitochondrial damage caused by calcium overload. This helps maintain neuronal viability, contrasting with the dysregulated calcium handling observed in neurodegeneration, which typically involves elevated ER calcium levels and impaired SOCE.

Early studies in polymorphonuclear (PMN) leukocytes demonstrated that trans-resveratrol (Table 1) inhibits the calcium release induced by formyl methionyl leucyl phenylalanine (fMLP) [78]. This inhibition was modest at 22 µM with near-maximal suppression observed at 110 µM of trans-resveratrol [78]. Platelets proved to be more sensitive to trans-resveratrol [46]. In these cells, trans-resveratrol inhibited thrombin-induced calcium release at a concentration as low as 0.1 µM, achieving a maximum inhibition of 57 ± 4% at 10 µM. Furthermore, trans-resveratrol effectively blocked Tg-induced calcium influx, implicating SOC channels as a potential molecular target. Notably, increasing extracellular calcium concentrations did not reverse the inhibitory effect of trans-resveratrol on thrombin-induced responses, suggesting that the polyphenol does not compete with calcium for a common binding site on the channel.

A well-known structural analog of resveratrol with inhibitory activity on calcium signaling is diethylstilbestrol (DES). DES effectively inhibited Tg-induced calcium influx in a dose-dependent manner with IC_50_ values of ∼0.1 μM in RBL cells, <0.1 μM in smooth muscle cells, and ∼1 μM in human platelets [79]. Despite its serious side effects, including teratogenicity and carcinogenicity, DES serves as a valuable tool for identifying the structurally active determinants of polyphenols. Although both compounds have a stilbene backbone, they differ in the number and position of hydroxyl groups. Resveratrol possesses three hydroxyl groups (with a 2-resorcinol or meta pattern on one aromatic ring), whereas DES has only two. Moreover, the ethyl groups in DES, attached via ether linkages, introduce steric bulk and alter the molecular conformation compared to the more compact and rigid trans-resveratrol. Critically, *trans*-stilbene, a close structural analog of DES that lacks both hydroxyl and ethyl groups, had no effect on SOCE [79], underscoring the essential role of these functional groups.

The importance of free phenolic hydroxyls is further highlighted by SAR studies. The monobenzyl ether of DES, in which one phenolic hydroxyl is blocked by a bulky benzyl substituent, is virtually inactive. Modification of the phenolic groups generally reduces efficacy of DES by 2- to 10-fold. These results strongly indicate that hydrogen bonding or other polar interactions mediated by free hydroxyl groups are critical for target engagement. However, the dipropionate and dimethyl ether esters of DES retain some activity, suggesting that not all ester substituents equally hinder binding.

The conformational rigidity of the stilbene moiety also plays a pivotal role. DES, which has a rigid, nearly planar structure due to the double bond, is a potent inhibitor. In contrast, *trans*-stilbene, which also has a double bond but lacks the DES side chains and another essential aromatic ring at positions 3 and 5, is inactive and does not affect calcium influx into platelets. This demonstrates that the mere presence of phenolic hydroxyls alone is insufficient—their orientation, relative positioning, and the fixed conformation of the molecule are equally important. Interestingly, side chains in tetrahydrochrysene (THC) derivatives have been proposed to block IP_3_-sensitive internal calcium release, while trans-resveratrol and genistein, which lack such side chains, exhibit inhibitory potencies comparable to DES and THC derivatives bearing aliphatic side chains.

Other polyphenols, including genistin, genistein, daidzein, and apigenin, exhibit a mechanism of action similar to that of trans-resveratrol on thrombin-stimulated platelets, inhibiting calcium influx in the concentration range of 0.1 ± 10 µM. Genistein and its more hydrophilic glycoside, genistin, suppressed calcium influx in a similar manner, suggesting that these polyphenols may act directly on the platelet surface to modulate calcium channel activity [46].

In a more targeted SAR study, Sadaghiani et al. [47] modified known polyphenols (biochanin A and genistein) to develop a flavonoid-related agent, AnCoA4 [47]. Biochanin A exhibited low-dose-dependent inhibition, and the removal of all methoxy groups (yielding genistein) almost completely abrogated activity. However, methylation and addition of an ethyl ester to a polyphenol derivative partially restored this activity [47]. Based on these results, the authors concluded that the core pharmacophore consists of a three-ring isoflavone-like structure. Furthermore, small methoxy groups on ring A and a phenyl moiety on ring C were identified as essential for inhibitory activity, whereas bulky substituents on ring B were detrimental. Thus, optimization of rings A and C proved promising, culminating in the synthesis of AnCoA4. To confirm that AnCoA4 directly inhibits Orai1 channels, the authors performed whole-cell patch-clamp recordings in HEK293 cells. Application of 20 μM AnCoA4 resulted in an 80% reduction in calcium currents [47]. Furthermore, AnCoA4 enhanced the fluorescence of the mCherry probe fused to the C-terminus of Orai1 but had no effect on probes at the N-terminus or on loop 2–3. This result was also confirmed by the surface plasmon resonance approach.

Evaluation of the FRED docking algorithm to search for binding sites on the Orai1 homodimer and potential intermolecular interactions between Orai1 and polyphenol derivatives highlighted with MacPyMol allowed the identification of the putative inhibitor-binding site. AnCoA4 was predicted to insert between the C-termini of adjacent Orai1 subunits, engaging in polar contacts with His299, Glu318, Thr312, as well as *π*-*π* and hydrophobic interactions with Phe300. Notably, overexpression of STIM1 almost completely abrogated AnCoA4-mediated channel inhibition, suggesting that STIM1 and AnCoA4 compete for an overlapping or identical site on Orai1.

Finally, quercetin and kaempferol have also been shown to inhibit SOCE [80]. Molecular docking revealed high binding affinity of these polyphenols to Orai, with hydrogen bonds forming to Ser82, Ile148, and Glu173 and additional aromatic and hydrogen bonds to Glu166 near residue Ser75, which is located in the channel pore region within TM1 in Orai1 [80].

### 3.4. Indazole Derivatives

Indazole is a bicyclic heteroaromatic system comprising a benzene ring fused to a five-membered pyrazole ring. The pyrazole moiety contains two adjacent nitrogen atoms and exists in tautomeric forms, of which 1H-indazole isomer is the most common and thermodynamically stable. The attachment of various functional groups to this core generates a diverse range of derivatives with significant biological and medicinal applications. Indole, a related bicyclic system, is recognized as a versatile pharmacophore due to its capacity to engage multiple receptor targets, yielding a broad spectrum of biological activities. As a result, it is regarded as one of the most privileged frameworks in medicinal chemistry [81]. Indazole derivatives, by virtue of their structural similarity, have similarly attracted considerable attention as modulators of calcium signaling.

Bai et al. [48] conducted a systematic SAR analysis of two series of indazole derivatives—3-aminoindazoles and indazole-3-carboxamides, both bearing an amide moiety and variable arylamine (Ar) substituents (Table 1). Their study assessed the inhibitory activity of these compounds on calcium influx in the rodent mast cell line RBL-2H3 and identified several key structural determinants for CRAC channel inhibition.

A critical finding was the profound influence of the amide linker orientation. Derivatives containing the direct amide linker (-CO-NH-Ar) exhibited significantly greater potency than most previously reported CRAC inhibitors, which typically feature the reverse linker (-NH-CO-Ar). The IC_50_ of the most potent 3-aminoindazole derivative with reverse amide linker (-NH-CO-Ar) is 29 μM [48]. The remaining compounds either close to 50% inhibition at 30 μM or were active only at 100 μM. In contrast, the IC_50_ of the most potent blockers, which contain direct linker, ranged from 0.67 to 1.51 μM. The nature of the Ar group also proved crucial for activity. Compounds bearing 2,6-difluorophenyl and 3-fluoro-4-pyridyl substituents exhibited the highest inhibitory potency. Conversely, the introduction of 2-chloro-6-fluorophenyl (IC_50_ = 3.23 μM) or 2,4-difluorophenyl (IC_50_ = 2.33 μM) groups reduced the modulatory effect of the compounds, while the presence of the 3,5-difluoro-4-pyridyl or 3-methyl-4-pyridyl groups completely abolished activity [48].

An alternative strategy for structural modification involves substitution at the N1 position of the indazole ring. The 2,4-dichlorobenzyl moiety has been identified as a key pharmacophoric element for inhibitory activity of these compounds. However, this group also contributes substantially to the high hydrophobicity of the inhibitors, which limits their use as pharmacological tools [48]. For instance, the indazole derivatives reported by Bai et al. [48] exhibited calculated *clogP* values (estimated using ChemAxon V14.8.18.0) ranging from 4.65 to 6.18 [48]. To address this limitation, Liardo et al. [82] replaced the highly lipophilic phenyl ring with bioisosteric cycloalkyl radicals, including *c*-propyl and *c*-butyl groups [83,84]. This substitution strategy successfully reduced lipophilicity: replacing the 2,4-dichlorobenzyl fragment with cycloalkyl moieties containing 3 to 6 carbon atoms lowered *clogP* to at least 3.60. The most promising compound in this series, 4k [1-(cyclopropylmethyl)-N-(3-fluoropyridin-4-yl)-1H-indazole-3-carboxamide], achieved a *clogP* of 2.52 [82]. Moreover, 4k completely blocked CRAC current (IC_50_ = 4.9 μM) and inhibited NFAT nuclear translocation at 30 and 10 μM, respectively, without affecting the electrophysiological function of TRPM4 and TRPM7 channels at concentrations up to 30 μM. To elucidate the binding mode of 4k with its primary target, Orai1, molecular modeling studies were performed [82]. The authors examined the 3D structures of *Drosophila melanogaster* Orai (dOrai), which shares 73% sequence identity with human Orai1, in both closed and open conformations in the presence of the 4k inhibitor.

In silico modeling suggested that 4k occupies the upper pore region of the Orai1 channel and forms two hydrogen bonds. The first interaction occurs between the amide –NH group of 4k and the carboxylate side chain of Glu106 (pKa = 7.90), while the second involves the pyridine nitrogen and protonated carboxyl side chain of the same Glu106 residue (pKa = 7.57). Hydrophobic interactions further stabilize the inhibitor–protein complex. The cyclopropyl group at the N1 position is predicted to reside in the hydrophobic pocket, engaging with the side chains of Phe99E/F and Val102F. Electrophysiological studies using patch-clamp recordings on Orai1 carrying the G98S mutation confirmed that the Orai channel is a direct target of these CRAC blockers, consistent with the computational predictions [82].

### 3.5. Imidazole Derivatives

Imidazole derivatives constitute a significant class of nitrogen-containing heterocyclic compounds characterized by a five-membered, planar, aromatic ring with three carbon atoms and two non-adjacent nitrogen atoms (positions 1 and 3). The aromaticity of the ring is conferred by a cyclic system of six *π*-electrons. The presence of two distinct nitrogen atoms endows the molecule with dual chemical reactivity: a pyridine-like N that confers basicity and a pyrrole-like N that provides acidic character. The structural diversity of this class is achieved through the introduction of various substituents onto the imidazole nucleus, yielding compounds with a broad spectrum of biological and industrial applications, including the endogenous signaling molecule histamine and numerous synthetic antifungal agents.

Imidazole derivatives have shown promise in the treatment of neurodegenerative diseases, such as AD, through a multi-target-directed ligand strategy. These multifunctional compounds can simultaneously combat oxidative stress, reduce inflammation, inhibit the aggregation of amyloid peptides (like A*β*), and modulate cholinesterase activity. Current research efforts are, therefore, directed toward optimizing the pharmacokinetic properties of these derivatives—particularly their ability to permeate the blood–brain barrier—and enhancing their biological activity profile to facilitate progression into clinical trials for neurodegenerative disorders.

The imidazole antifungal agent SKF-96365 (1-{*β*-[3-(p-methoxyphenyl)-propyloxy-p-methoxyphenylethyl]}-1H-imidazole hydrochloride) was among the first experimental inhibitors of CRAC channels to be identified (Table 1) [85]. This compound has been demonstrated to block agonist-induced calcium influx in various cell types, including rat basophilic leukemia (RBL), mast cells, Drosophila S2, and Jurkat T cells, at concentrations typically ranging from 10 to 30 μM [85]. Specifically, using patch-clamp and indole-1 fluorescence assays on the Jurkat T leukemia cell line, Chung et al. [49] showed that SKF-96365 dose-dependently inhibited Tg-mediated *I_CRAC_* with an IC_50_ of 12 μM.

Beyond SKF 96365, other imidazole antifungals, including econazole, miconazole, and clotrimazole, have been also shown to inhibit CRAC currents with IC_50_ values ranging from 0.56 to 3 μM [86]. For instance, Christian et al. [86] demonstrated that econazole at its highest tested concentration (30 μM) produced 71 ± 11% inhibition of *I_CRAC_* (*n* = 13) in a human T-lymphocyte-derived cell line. Despite their efficacy in inhibiting CRAC channels, imidazole derivatives are hindered by a lack of selectivity. At comparable potencies they also suppress the activity of other ion channels, including voltage-gated calcium channels, non-selective cation channels, and cyclic AMP-gated chlorine channels [86]. This off-target activity, partially evident with econazole and miconazole [86], has limited their further development and clinical utility as specific CRAC channel modulators.

A central aspect of the design of the new compounds was the replacement of the imidazole ring (present in SKF-96365) with a pyrazole ring, resulting in increased inhibitory activity (reducing in IC_50_) against SOCE. The study demonstrated that even a minor modification of the heterocyclic scaffold, specifically, changing N-3 in imidazole to N-2 in pyrazole, exerts a more potent inhibitory effect on SOCE (reducing the IC_50_ from 60 to 25 μM for 7d (1R, 1S) 1-[b-((4-Methoxyphenyl)-(3-(4-methoxyphenyl)propoxyethyl)]-1H-pyrazolium hydrochloride))) [87].

SAR analysis identified two key factors determining activity. First, the presence or absence of a methoxy group on the phenylethyl scaffold or in the phenyloxy side chain significantly modulates the IC_50_ value [87]. The absence of a methoxy group on the pyrazole derivative’s scaffold enhances its inhibitory potency (reducing the IC_50_ from 34 to 25 μM). Conversely, the absence of this group in the side chain reduces the potency of imidazole derivatives (increasing the IC_50_ to 48 μM). Second, the length of the side chain connecting the phenylethyl core and the phenyloxy group is a critical parameter. Extending the linker to a propylene chain improves activity compared to shorter analogs, such as those with an ethylene linker (IC_50_ = 48 μM) [87]. The modifications performed demonstrated higher selectivity for SOCE (IC_50_ = 25 μM for 7d vs. 60 μM for SKF-96365) with a reduced effect on intracellular Ca^2+^ release. Thus, modifying the SKF-96365 structure by replacing the imidazole with a pyrazole and introducing methoxy groups onto the phenylethyl scaffold may lead to the development of more potent SOCE inhibitors.

Research into the mechanism of action of imidazoles is extremely limited in the literature. It is known that Christian et al. [86] used standard whole-cell patch-clamp recording techniques to study the pharmacological characteristics and site of econazole-mediated inhibition of calcium-release-activated calcium current in the human leukemia T-cell line, Jurkat. The authors proposed that econazole may exert its blocking effect from an extracellular rather than intracellular site, suggesting a direct interaction with an extracellularly accessible domain of the channel protein.

### 3.6. Indole Derivatives

The indole core, a weakly basic structure comprising a pyrrole ring fused to a benzene ring, is characterized by its aromaticity, which arises from delocalization of ten π-electrons. Indole derivatives have long fascinated researchers due to their diverse biological activities and substantial therapeutic potential. As core structural motifs in numerous natural products and pharmaceuticals, indoles serve as pivotal scaffolds in drug discovery and development.

In a search for novel CRAC channel antagonists, Kong et al. [88] screened a commercial library of chemical compounds (Selleck Chemicals LLC (Houston), Texas, TX (Houston), USA; catalog number L3600) [88] and identified a new indole-like compound, C63368 ((1Z)-1-(1,3,3,5-tetramethyl-1,3-dihydro-2H-indol-2-ylidene)propan-2-one). The authors evaluated its ability to inhibit *I_CRAC_* (at a concentration of 10 μM) in HEK293T cells co-expressing STIM1 and Orai1. The IC_50_ value of C63368 required for *I_CRAC_* inhibition was 2.2 ± 0.3 μM. In a selectivity panel, most ion channels were resistant to C63368; however, the KV1.3 channel was the most sensitive, exhibiting approximately 38% inhibition with an estimated IC_50_ of 131.3 ± 2.6 μM.

Selectivity testing of C63368 against a panel of voltage-gated sodium, potassium, and calcium channels, as well as TRPM4 and TRPM7 channels, at a single dose of 100 µM revealed that the majority of the ion channels were resistant to the compound’s action. The KV1.3 channels exhibited the highest sensitivity to C63368 (37.9 ± 0.03%), with an IC_50_ of 131.3 ± 2.6 µM. Analysis using the Hill equation confirmed the compound’s high selectivity for the CRAC channel. The selectivity range for C63368 extended from 60-fold (relative to KV1.3) to 4500-fold (relative to CaV3.1).

Using 7-azaindoles as a model system, Esteve et al. [50] described the synthesis and pharmacokinetic properties of indole-like inhibitors of Orai channels. The authors initially performed substitutions at position 2 of the 7-azaindole core via a single alkyl bond. Their SAR studies revealed that compounds bearing a 2,6-disubstituted phenyl group exhibited the most potent inhibitory activity against CRAC channels in Jurkat cells [50]. Specifically, the compound 9b (2-chloro-5-fluoro-N-(1-methyl-3-(3-(trifluoromethyl)phenyl)-1H-pyrrolo [2,3-b]pyridin-5-yl)benzamide), substituted with 2-chloro-6-fluorophenyl group, demonstrated an IC_50_ of 66 nM in a fluorescence-based assay measuring SOCE. However, the introduction of polar groups or benzyl groups led to a sharp decline in inhibitory activity.

Consequently, the authors varied the disubstituted aryl, heteroaryl, and bicyclic groups, at position 5, while maintaining the 2-chloro-6-fluorophenyl moiety at position 2. Compounds featuring disubstituted 3-pyridyl groups (derivatives 14a–d) yielded the highest calcium inhibition potency in Jurkat cells, with IC_50_ values ranging from 64 to 150 nM. In contrast, the introduction of more polar substituents, such as N-methylpyridone or hydroxypyridine groups at position 5, resulted in a complete loss of activity. Replacing the pyridine ring of compound 14b with a pyrimidine similarly diminished activity, as did the introduction of basic groups.

A similar trend was observed for sulfonamide derivatives, N-methyl and N,N-dimethyl 3-methylbenzenesulfonamides, as well as the sulfone-containing derivative retained acceptable activity, whereas the sulfonylmorpholine derivative was inactive. Substitution at R1 with bicyclic groups, such as 6-chlorobenzoxazole and 6-chloro-2,2-difluorobenzodioxolyl groups (derivatives 14m–n), was also tolerated. However, these molecules exhibited very high lipophilicity (*clog* *P* > 5) and low solubility (<1 μg/mL) [88].

Ultimately, the authors selected the most biologically active derivative, 14d—a 2-chloro-5-fluorophenyl derivative of 7-azaindole with an N-methylpiperidine substituent at position 3 of the azaindole core—for further testing [50] (Table 1). Compound 14d exhibits marked functional selectivity for Orai1 calcium channels in T-lymphocytes. In Jurkat cells, it inhibits calcium influx (IC_50_ = 150 nM) and IL-2 secretion (IC_50_ = 68 nM). However, its activity in LAD2 mast cells is 4–10 times lower (IC_50_ = 640 nM), indicating a preferential effect on the T-cell phenotype of Orai1. The compound has little effect on alternative degranulation pathways (RBL-2H3, IC_50_ > 10 µM) and is virtually devoid of cytotoxicity (IC_50_ > 100 µM in CHO cells). Thus, 14d is a highly selective Orai1 inhibitor, making it a valuable tool compound for studying the role of SOCE in the pathogenesis of inflammatory and allergic diseases.

Kong et al. [88] further demonstrated that the mechanism of CRAC channel inhibition by these indole-like compounds involves direct blockade of the channel pore, confirmed by whole-cell patch-clamp recordings showing rapid, reversible inhibition of Orai1 currents and the lack of effect on Ca^2+^-dependent inactivation. This conclusion is supported by the observation that multiple mutations in the outer pore region reduced the inhibitory potency of the compounds. Moreover, FRET and confocal imaging experiments ruled out the possibility that these compounds inhibit the CRAC channel by interfering with STIM1 activation or STIM1–Orai1 coupling. Notably, the inhibitory efficacy was reduced against the Orai1/V102A and Orai1/261 ANSGA265 mutant channels compared to wild-type Orai1. Mutation of several pore-lining residues in the Orai1 subunit, that increase pore diameter (such as triple mutations E106D and D110A/D112A/D114A), also significantly weakened the inhibitory effect [89]. These residues likely either directly contribute to the formation of the compound-binding pocket or, when mutated, allosterically alter the pore geometry, reducing compound affinity. Additionally, mutation of residue E190 in the TM3 helix of Orai1, which also increases pore size and alters their geometry, further supports this mechanism.

It has been previously reported that V102 functions as a hydrophobic gate, and Orai1/V102A forms a constitutively leaky channel in the resting state [90,91]. C63368 and C79413 ((5Z)-3-(1,5-dimethyl-3-oxo-2-phenyl-2,3-dihydro-1H-pyrazol-4-yl)-5-(4-hydroxybenzylidene)-2-thioxo-1,3- thiazolidin-4-one) effectively inhibited this current mediated by the Orai1/V102A mutant, as measured via whole-cell patch-clamp in Ca^2+^-free extracellular solution, supporting a direct effect on the Orai1 protein. Collectively, these data integrating the results from site-directed mutagenesis, patch-clamp pharmacology, and fluorescence-based interaction assays suggest that the compounds bind within the channel pore, where V102 may directly participate in the binding site, or, alternatively, the V102A mutation may allosterically modify the pore architecture, thereby reducing compound binding.

### 3.7. Benzamide Derivatives

Among the numerous classes of compounds investigated as CRAC channel modulators, benzamides have gained prominence due to their structural versatility and pharmacological potential. Benzamide derivatives typically consist of a substituted benzene ring directly linked to an amide group, providing a versatile and stable scaffold in drug design. This motif is widely employed in neuroleptics, anti-emetics, and anticancer agents and is also found in several SOCE inhibitors. A number of structurally related compounds are currently promising candidates for clinical trials. For instance, PRCL-02 (also known as STA-12-8336), an Orai1 inhibitor developed by PRCL Research Inc., was proposed for a phase I trial in patients with psoriasis (NCT03062618). The drug was subsequently advanced to a phase IIa clinical trial (NCT03614078) to evaluate its safety, tolerability, and efficacy in patients with moderate to severe chronic plaque psoriasis [11]. However, limited public data are available for this compound, and its specific in vitro IC_50_ has not been disclosed in the literature. The most extensively studied agents with a structure similar to PRCL-02 are CM4620 and Synta66, which have attracted considerable attention as promising SOCE modulators (Table 1).

CM4620, also known as zegocractin (Auxora), is a selective Orai1 inhibitor, being investigated as a potential therapeutic agent for acute pancreatitis (Table 1) [52,92]. In vitro studies demonstrate that CM4620 effectively inhibits SOCE in pancreatic acinar cells and reduces intracellular calcium concentrations, which may confer a protective effect in the context of pancreatitis. Lewis et al. [52] reported that submicromolar concentrations of CM4620 significantly inhibit calcium influx into FACS (fluorescence-activated cell sorting) in the concentration range of 1–100 nM. CM4620 was reported to be approximately 7-fold more potent at inhibiting Orai1/STIM1-mediated calcium currents compared to Orai2/STIM1-mediated currents, as judged by IC_50_ values of approximately 120 nM and 900 nM, respectively [93]. Synta66, according to Waldherr et al. [94], directly blocks the Orai1 channel, with high selectivity. Its efficacy in blocking SOCE was demonstrated in glioblastoma cell lines, with an IC_50_ of ≈4 μM. Both compounds serve as valuable pharmacological tools for studying and modulating calcium signaling in various physiological and pathological processes.

It is important to note that in biological environments, Synta66 is susceptible to amide group hydrolysis, which reduces its metabolic stability. For example, in rat liver microsomes, only about 15% of the parent compound remains after an hour of incubation [94]. Furthermore, according to PubChem, CM4620 exhibits increased lipophilicity, which may impair its bioavailability. Therefore, attempts have been made to investigate and improve some of the pharmacokinetic properties of benzamide-based inhibitors. Notably, many benzamide derivatives studied to date feature a 2,6-difluorobenzamide core.

Khedkar et al. [95] demonstrated that fluorinated phenyl groups at positions 2 and 4, or 2 and 5, on the benzamide ring were inactive. However, replacing fluorine with chlorine at position 2 yielded an IC_50_ comparable to that of CM4620 and Synta66 (171 nM), and combining this with fluorine at position 6 led to a significant improvement, with an IC_50_ of 65 nM. Similarly, replacing the substituted benzamide moiety with unsubstituted 4-pyridyl resulted in an inactive compound, whereas the introduction of a 3,5-difluoropyridyl group restored significant inhibitor activity (IC_50_ ≈ 95 nM), highlighting the critical role of fluorination [95].

The position and nature of the heterocyclic ring A play a pivotal role in modulating biological activity. Positions C3 and C5 in the phenyl moiety were found to be more favorable for maintaining activity compared to position C4. Increasing polarity by introducing specific heterocycles also positively influenced activity [95]. For instance, incorporating a five-membered heterocycle (5,5-dimethyl-4-oxo-4,5-dihydroisoxazol-3-yl) as ring A at position C5 relative to ring B resulted in a decrease in lipophilicity (*clogP* ranged from 2.88 to 4.58). In addition, the authors demonstrated a dependence of activity on the size and electronic properties of substituents on the phenyl ring B. Smaller alkyl and alkoxy groups were found to be more favorable for inhibiting SOCE (IC_50_ = 345 and 379 nM, respectively) than bulky tert-butyl substituents, which resulted in a loss of activity [95].

A study of bioisosteric linkers revealed that the “reverse” amide (-CO-NH-Ar) resulted in reduced activity (IC_50_ = 150 nM), whereas urea and sulfonamide linkers suppressed activity almost completely. Thus, both the amide and “reverse” amide linkers were deemed acceptable, but the amide was selected for further SAR optimization due to its higher efficacy [95].

Following optimization of rings, A, B, D, and the linker, the effect of ring C on activity was studied, while keeping the optimized rings fixed. Removal of one nitrogen atom from ring C (pyridine analog) decreased activity, whereas removal of both nitrogen atoms (phenyl analog) preserved it. Replacing the pyrazine ring C with a phenyl ring resulted in a significant improvement in SOCE inhibition (IC_50_ = 140 nM). The phenyl ring C also provided a better pharmacokinetic profile (area under the curve (AUClast) = 75 h · μM) compared to the pyrazine analog [95].

A systematic SAR study aimed at optimizing the balance of lipophilicity and polarity successfully identified two lead compounds, 36 (2,6-difluoro-N-(2′-methyl-3′-(4-methyl-5-oxo-4,5-dihydro-1,3,4-oxadiazol-2-yl)-[1,1′-biphenyl]-4-yl)benzamide) and 37 (2-chloro-6-fluoro-N-(2′-methyl-3′-(4-methyl-5-oxo-4,5-dihydro-1,3,4-oxadiazol-2-yl)-[1,1′-biphenyl]-4-yl)benzamide). Both compounds exhibited significantly improved plasma exposure (AUClast = 353 and 167 h · μM). They were metabolically stable in liver microsomes from multiple species and did not exhibit significant inhibition against eight major cytochrome P450 isoforms [95]. The compounds 36 and 37 demonstrated high selectivity for SOCE and were virtually devoid of activity against voltage-operated calcium entry; furthermore, their effects on NF-κB and AP-1 were strictly mediated by the blockade of calcium entry and did not involve calcium-independent activation pathways. The lack of effect on cytochrome P450 makes these molecules not only highly specific but also safe candidates for the treatment of autoimmune diseases [95].

Khedkar et al. [95] predicted the putative binding site by constructing a homology model of the Orai1 channel and docking compound 36 into this model. The compound was shown to bind to Orai1 in a region very close to the STIM1–Orai1 interaction interface, locking the channel in an inactive conformation and preventing STIM1 binding. Furthermore, the glutamic acid residue at position 106 was identified as critical for compound binding and functional activity.

A somewhat different mechanism was described for Synta66. It has been shown that Synta66 inhibited CRAC channels with equal efficacy whether administered extracellularly or intracellularly [43]. Using docking and molecular dynamics simulations, Waldherr et al. [94] demonstrated that Synta66 binding is localized to an extracellular region near transmembrane helices TM1 and TM3, as well as extracellular connecting loop segments 1 and 3 adjacent to the hOrai1 selectivity filter [94]. Analysis of the docked complex using the protein–ligand interaction profiler (PLIP) revealed several key interactions between the Synta66 ligand and the putative extracellular inhibitor-binding site on the rOrai1 protein. In particular, hydrogen bonds are formed between the methoxy group of the terminal aryl moiety and Asp110, as well as between the amide group and Glu106. Hydrophobic contacts are established between the intermediate aryl moiety and Val107, and between the fluoropyridine fragment and Glu106 in hOrai1 [43].

The most favorable intracellular binding site for Synta66 was identified as a pocket near the M3 and M4 helices of adjacent Orai1 subunits. PLIP revealed interactions between the central aryl group and His256 and between the fluoropyridine moiety and Phe267, as well as a series of hydrophobic contacts involving Pro146, Leu174, Phe253, Glu272, and Leu276 in this region of hOrai1 [43].

Furthermore, the glutamic acid residue at position 106, located in the pore-forming M1 helix, was identified as critical for inhibitor binding, selectivity, pore diameter, and pore hydration. Specifically, it was found that increasing the hydration of the Orai1 pore (whether by using mutant variants of Orai1 loop 1 (L109D, H113G, and Y115G) and loop 3 (F199G, P201G, and L202G), or by substituting His134 with Ala (Orai1-H134A) and Glu106 with Asp (Orai1-E106D)) leads to a decrease in selectivity and a weakening of the blocking effect of Synta66 on the channel [43]. This is further supported by experiments replacing extracellular calcium with sodium, characterized by lower hydration. The sodium currents were shown to be only partially inhibited by Synta66, whereas complete inhibition was observed in calcium-containing solutions. Thus, ion hydration within the Orai1 selectivity filter region plays a crucial role in the mechanism of channel inhibition by Synta66.

### 3.8. Triazole Derivatives

Triazoles are a class of heterocyclic compounds with the molecular structure, featuring a five-membered aromatic ring containing three nitrogen atoms. These compounds primarily exist as two main isomeric forms—1,2,3-triazole and 1,2,4-triazole—forming the core scaffold for numerous bioactive molecules, pharmaceuticals, and agricultural chemicals. Triazoles are valued for their stability, capacity for hydrogen bonding, and ability to engage in π-π interactions, as well as their favorable pharmacokinetic profiles, making them attractive candidates for bioactive compound development.

Carboxyamidotriazole (also known as CAI) is a synthetic triazole derivative employed in biomedical research as a calcium channel inhibitor and antitumor agent (Table 1) [96]. CAI has been shown to suppress transmembrane calcium flux and disrupt intracellular calcium signaling, primarily in non-excitable cells, including endothelial and tumor cells [53,97,98,99,100]. This activity may result in multiple inhibitory effects on processes associated with tumor growth and angiogenesis. Specifically, CAI reduces the production of basic fibroblast growth factor (bFGF) and matrix metalloproteinase-2 (MMP-2) and suppresses vascular endothelial growth factor (VEGF)-stimulated cell proliferation and migration [53,100]. Additionally, CAI inhibits the secretion of both VEGF and the proinflammatory cytokine interleukin-8 (IL-8) by tumor and endothelial cells [99].

CAI, whose mechanism involves, among other actions, inhibition of calcium homeostasis, was investigated as monotherapy in patients with advanced, metastatic stage III renal cell carcinoma (RCC) who had previously received immunotherapy. Although CAI has entered clinical trials, this compound suffers from limited oral bioavailability, significant gastrointestinal and neurotoxicity, and low metabolic stability [53,101]. These limitations have necessitated SAR studies and the development of new CAI derivatives. Efforts to optimize the hydrophobicity, metabolic stability, and solubility of CAI analogs required the identification of the functional roles of key molecular fragments. Initially, the molecular structure of CAI comprises three key elements: a triazole ring, a benzophenone bridge, and a methylene group, linking the triazole ring to the central benzene moiety. The triazole moiety bears an amino group at position 5 and a carboxamide group at position 4. The benzophenone bridge comprises a central phenyl ring substituted with two chlorine atoms at positions 3 and 5, and a chlorobenzene moiety bearing a carbonyl group. Dehalogenation of CAI or truncation of the benzophenone moiety resulted in significant or complete loss of activity [53], highlighting the importance of a fully substituted benzophenone moiety for maintaining the required tertiary conformation and target interaction. Removal of either the substituted benzophenone or the triazole group also reduced the compound’s potency, indicating that both moieties are essential for calcium influx inhibition [53].

To enable greater structural variation and to modify and improve the pharmacological properties of triazole derivatives, agents with more complex architectures were selected. According to Riva et al. [40] and Serafini et al. [54], the biphenyl system offers more opportunities for substituent variation and optimization compared to the simple aromatic ring used in earlier studies [40,54]. Consistent with Kohn et al. [53], these studies emphasize the importance of the triazole ring for activity, where the triazole is incorporated into pyrtriazole and biphenyltriazole skeleton. Riva et al. [40] noted that the common arylamide moiety may represent an integral pharmacophoric feature of many non-selective SOCE inhibitors [40]. For example, such a structure is present in pyrazole derivatives, including BTP2 compounds and Pyr series analogs. To increase selectivity, the authors isosterically replaced the amide moiety with a 1,4-disubstituted 1,2,3-triazole ring. The same approach was used by Serafini et al. [54] using Synta66 as a reference.

The most variable part in both groups of compounds is the functional radical at the N3 or C4 position of the triazole ring [54]. Both studies agree that the introduction of a carboxyl group (-COOH) at the *meta*- or *para*-position of the phenyl ring conjugated with the triazole ring often enhances inhibitory activity and provides high water solubility in the sodium salt form [54].

However, in pyrtriazoles, carboxyl moieties at these positions can lead to either positive or negative modulation of SOCE, depending on the substitution pattern of the pyrazole moieties and neighboring substituents [40]. The COOH-group can be present in both highly active and less active agents. Furthermore, replacing the COOH-group with chlorine radical at the *para*-position also imparts a positive modulatory character to the molecule. Modification of the pyrazole fragment in pyrtriazoles revealed that the CF_3_ groups at the C3 and C5 positions of the pyrazole ring, or alternatively, the ethyl carboxylate group at the C4 position, are required for inhibitory activity and remain fixed. Additionally, methyl, isopropyl, or pentan-2-yl esters at the C4 position of the pyrazole ring were more favorable than ethyl esters (compound 39 (3-(1-(4-(4-((pentan-2-yloxy)carbonyl)-5-(trifluoromethyl)-1H-pyrazol-1-yl)-phenyl)-1H-1,2,3-triazol-4-yl)benzoic acid)) [40]. Riva et al. [40] compared the selectivity of the most promising synthesized compound, triazole derivative 39, with well-known pyrazoles Pyr3 and Pyr6 regarding the voltage-gated calcium channels TRPV1 and TRPM8 at a concentration of 10 μM. It was found that, unlike the pyrazoles, compound 39 had no effect on these channels [40].

Since pyrtriazoles exhibited highly variable activity, and the pyrazole ring limited the framework for compound modification, research subsequently focused on modifying the biphenyl moiety at the N1 position of the triazole ring [54]. The authors introduced methoxy groups into the molecules, which can render the compound less susceptible to enzymatic degradation, thereby increasing metabolic stability. Removal of methoxy groups from the 2′ and 5′ positions or the presence of only the 2′-methoxy group led to a significant decrease in activity. The presence of a methoxy group at the 4′ position increased inhibition efficiency to 50%, whereas a methoxy group at the 6′ position resulted in decreased activity. Compounds bearing a methoxy group at the 3′ position, instead of the 2′ position, showed high activity, reaching 85–96% [54]. Notably, compounds, with a 2′,3′-dimethoxyphenyl substituent, particularly compound 34, exhibited the best results (93–97% inhibition). Replacing the methoxy group with a hydroxyl or thioether group decreased the activity. Combining methoxy groups into a 1,4-dioxanyl ring led to a reduction in activity (up to 44% decrease). The 3′,5′-dimethoxyphenyl substituent showed inhibitory activity of approximately 74%, whereas methoxy groups at the 3′ and 4′ positions conversely decreased inhibition (to an average of 47%). Combining these substituents into a 1,4-dioxanyl ring increased the activity by 1.5-fold, while the agent bearing a 1,3-dioxolanyl ring was inactive. In turn, the 2′-fluoro-5′-methoxyphenyl ring provided high activity, ranging from 75 to 89% [54].

As a result, the authors identified compound 34 (3-(1-(2′,3′-dimethoxy-[1,1′-biphenyl]-4-yl)-1H-1,2,3-triazol-4-yl)-benzoic acid), which, compared to Synta66, exhibited slightly reduced activity against SOCE (IC_50_ = 851 ± 54 nM vs. 228 ± 33 nM), but was significantly less cytotoxic in HEK293 cells and demonstrated substantially higher metabolic stability in vitro (75% vs. 15% residual substrate after 1 h; Table 1). Furthermore, the presence of a carboxyl group in the inhibitor molecule ensured high aqueous solubility in the sodium salt form (1528 µg/mL vs. 0.28 µg/mL). This combination of properties afforded a favorable pharmacokinetic profile (following intravenous administration) and efficacy in a mouse model of caerulein-induced apoptosis.

## 4. STIM-Associated Small-Molecule Inhibitors

### 4.1. Diphenylborate Derivative 2-APB

Among the very limited repertoire of STIM inhibitors, 2-APB is the most well-known, extensively studied, and widely used compound. As noted earlier, its primary target is the Orai channels. However, it is suggested that the inhibitory effect of 2-APB may be mediated at both the channel and STIM1 levels.

A key mechanism of 2-APB inhibition involves the disruption of STIM1 puncta formation—the aggregates that form near the plasma membrane following the depletion of ER calcium stores. Using confocal microscopy, DeHaven et al. [45] demonstrated the distribution of STIM1, linked to yellow fluorescent protein, in Tg-free and Tg-treated HEK293 cells, both in the absence and presence of 50 μM 2-APB. The authors showed that 2-APB both prevents and reverses the reorganization of STIM1 into puncta. Goto et al. [69] also confirmed this effect for the diphenylborinate analogue of 2-APB DPB162-AE. This action is associated with enhanced intramolecular interactions between the coiled coil domain 1 (CC1) and STIM–Orai activating region (SOAR) of STIM1, which stabilizes the protein in its inactive state [55]. However, the ability of 2-APB to inhibit puncta formation is reduced when STIM1 is co-expressed with Orai1, indicating a complex interplay between these proteins.

Wei et al. [55] used FRET to find that 2-APB does not affect the dimeric state of STIM1 but enhances the intramolecular interaction between CC1 and SOAR of the STIM1 protein. CC1, in its coiled-coil form, acts as an autoinhibitory lock by binding to the SOAR domain, thereby maintaining STIM1 in an inactive state and preventing SOAR from engaging its target, the Orai1 channel.

### 4.2. Polyphenol Resveratrol

As previously described in this review, the mechanism of calcium entry inhibition by resveratrol is multifactorial, and Orai1 may represent only one of several targets. In particular, Lu et al. [102] reported that resveratrol decreased SOCE in a rat adjuvant arthritis model by affecting the Orai1/STIM1 dimer. Using co-immunoprecipitation with Orai1 or STIM1 antibody, the authors demonstrated that SOCE suppression correlated with reduced Orai1 expression and a decrease in the Orai1–STIM1 dimer, without significantly altering STIM1 expression levels. Furthermore, Orai1 overexpression restored the resveratrol-induced reduction in SOCE.

Previous studies have also reported that resveratrol can inhibit STIM1 binding to Orai1 by suppressing STIM1 phosphorylation in HEK293T cells, leading to downregulation of SOCE [56]. Casas-Rua et al. [56] calculated an IC_50_ of 10 μM in Fura-2-loaded HEK293 cells, overexpressing Flag–STIM1. The results of intracellular calcium measurements using the fluorescent probe Fura-2 showed that resveratrol did not significantly inhibit calcium release from the ER within the 0–50 μM range. Furthermore, it was shown that resveratrol did not directly inhibit the calcium channel pore, as simultaneous addition of resveratrol and calcium did not result in SOCE inhibition via pore blockade. Based on quantification of STIM1–EB1 co-immunoprecipitation by blot densitometry the authors hypothesized that resveratrol might inhibit ERK1/2 activation induced by calcium depletion, which in turn impedes STIM1 phosphorylation and dissociation of the STIM1–EB1 complex. This conclusion is supported by the lack of SOCE suppression in cells expressing the mutant STIM1-S575E/S608E/S621E or STIM1-I644N/P645N, mutations that prevent STIM1 dissociation from EB1, in the presence of resveratrol.

### 4.3. Steroid Hormone 17β-Estradiol

Also known as estradiol, 17*β*-estradiol (E2) is a steroid female sex hormone and is the major and most active estrogen in the human body (Table 1). Among other functions, this steroid mediates rapid non-genomic calcium-signaling pathways involving cell membrane receptors that activate intracellular cascades, leading to calcium influx through calcium channels. In particular, Coakley et al. [103] reported that E2 inhibits agonist-mediated calcium signaling through estrogen receptor α (ESR1) in a non-genomic manner in airway epithelia, which prevents the activation of calcium-activated chlorine channels and leads to airway dehydration [103].

Fluorescent measurements of STIM1 using actin counterstaining as a polarity marker showed that the subapical concentration of STIM1 was significantly reduced following E2 exposure, suggesting that E2 disrupts normal STIM1 function. These results correlated with a decrease in the FRET signal of the STIM1–STIM1 interaction for HEK293T cells pretreated with E2 [57]. Furthermore, in the presence of E2, STIM1 puncta formation at the ER–plasma membrane junction was significantly decreased following ER calcium depletion, indicating impaired oligomerization. The authors also investigated the role of STIM1 phosphorylation in E2-mediated inhibition of STIM1 mobility [57]. Using immunoprecipitation coupled with Western blotting against phosphoserine, and fluorescence recovery after photobleaching analysis of the STIM1-S575A mutant, E2 treatment significantly reduced phosphorylation of Ser575 in the C-terminal domain of STIM1, a residue known to influence protein function. Substitution of Ser575 with Ala inhibited STIM1 phosphorylation, reduced its translocation along microtubules, and decreased STIM1 sensitivity to E2. These data suggest that E2 may signal non-genomically by inhibiting basal phosphorylation of STIM1, leading to a reduction in SOCE.

### 4.4. Indole-3-Carbinol Derivative Femindol

The compound 3,3′-diindolylmethane (DIM) is an indole-3-carbinol derivative found in cruciferous vegetables and acts as an inhibitor of several key enzymes and receptors. Specifically, DIM can activate the STIM1-mediated SOCE signaling pathway in breast, glioma, and lung cancer cells with an IC_50_ ranging from 10 to 50 μM, leading to cell death by depleting ER calcium stores and increasing STIM1 levels [104]. Although DIM is a natural product, it has several significant drawbacks, such as poor bioavailability and a high effective concentration. However, a DIM analog with a substituted arylmethyl ring, called femindol, exhibits significant anticancer activity in human triple-negative breast cancer (TNBC) cells (Table 1). Chakraborty et al. [58] demonstrated that femindol exhibits in vitro activity two orders of magnitude greater than that of DIM in suppressing the tumor cell proliferation. This was assessed using the MTT viability assay, which revealed an IC_50_ of ~52 μM for femindol compared to ~72 μM for DIM. Femindol was found to regulate SOCE by reducing STIM1 protein levels in TNBC cells, as confirmed by both immunofluorescence staining and Western blotting. Unlike STIM1, treatment with femindol did not significantly affect Orai1 protein expression levels. Co-immunoprecipitation experiments showed that femindol inhibited the interaction between STIM1 and Orai1. The authors suggest that femindol slows STIM1 transport from the ER to the plasma membrane, where it interacts with Orai1. Moreover, STIM1 overexpression achieved via transfection with a STIM1-pEGFP plasmid restores the femindol-induced decrease in cell survival by inducing SOCE-dependent proliferation. These results indicate that femindol targets STIM1, altering SOCE in TNBC cells to exert its anticancer effect [58].

### 4.5. 5-Methyl-2-Phenyl-2H-1,2,3-Triazole-4-Carboxylic Acid (4-MPTC)

Recently, a library of 250 compounds obtained from InterBioScreen Ltd. (Chernogolovka, Russia). was screened to identify a selective modulator of the STIM2 protein using a Fura-2 AM-based calcium imaging assay in HEK293T cells co-expressing STIM2 and Orai1. This analysis identified 4-MPTC, which contains a thiazole ring with several substituents, including a pyridin-4-yl group, a pyridin-2-yl group, and a methyl group (Table 1). Here, 4-MPTC was identified as an inhibitor of SOCE, acting as a selective inhibitor of the STIM2-dependent SOCE pathway, with an IC_50_ of 1 µM [59]. Importantly, 4-MPTC did not reduce Orai3 channel activity. In STIM1 knockout cells, where STIM2 is the sole activator of SOCE, 4-MPTC effectively reduced the Tg-induced calcium response. In contrast, in cells where SOCE is predominantly activated by STIM1 (e.g., STIM2 knockout cells), 4-MPTC did not reduce the amplitude of the calcium response. While 4-MPTC modulates STIM2-mediated activity, its exact target has not been definitively identified yet.

### 4.6. Aminoglycoside Antibiotic G418

G418 (geneticin) belongs to the aminoglycoside class of antibiotics—a group of natural and semi-synthetic compounds characterized by an aminocyclitol core (2-deoxystreptamine), linked to amino sugars via glycosidic bonds (Table 1). This class includes well-known antibiotics such as gentamicin, neomycin, kanamycin, amikacin, and paromomycin. G418 itself is a 2-deoxystreptamine derivative substituted at positions 4 and 5 with two amino sugars: 2-amino-2,7-dideoxy-D-glycero-D-gluco-heptopyranose and 6-amino-6-deoxy-D-glucose.

Parvez et al. [60] first established that G418 acts as a highly specific and potent inhibitor of STIM2-mediated CRAC channel activation. This was demonstrated by recording currents in the whole-cell mode in HEK293 cells co-expressing STIM1/Orai1 or STIM2/Orai1, with intracellular delivery of G418 (10 µM) via the patch-pipette solution [60]. Investigating HEK293 cells expressing STIM1 or STIM2 coupled with Orai1, the authors observed a striking selectivity: intracellular administration of 10 μM G418 did not affect the currents of the STIM1/Orai1, but completely suppressed those mediated by STIM2/Orai1 [60], pointing to a unique binding site for G418 on STIM2 that is absent in the STIM1. The IC_50_ was approximately 0.6 μM with a Hill coefficient of 3, indicating cooperative binding of the antibiotic to the STIM2 [60]. Furthermore, while intracellular administration of G418 completely suppressed the currents, equimolar extracellular application caused only slight (approximately 20%) suppression, confirming the intracellular localization of the binding site [60].

Leveraging G418 as a specific pharmacological tool, Vigont et al. [61] confirmed the pivotal role of STIM2 in the pathogenesis of juvenile Huntington’s disease. Application of 10 μM G418 delivered intracellularly via the patch pipette in whole-cell patch-clamp recordings of primary HD76 neurons reduced the amplitude of SOCE currents in these ones to levels comparable to those in wild-type cells, as shown by measuring thapsigargin-induced Ca^2+^-selective currents [61]. This finding confirmed that STIM2 is the key driver of pathological hyperfunction of CRAC channels in neurons specific for the juvenile form of Huntington’s disease.

## 5. Other Compounds with Inhibitory SOCE Activity

This review examines the major widely studied inhibitor classes, focusing on compounds with chemically related structures. It addresses SAR and, where sufficient literature data exist, explores the possible mechanisms of action for these compounds. However, it should be noted that many newly synthesized, low-potency compounds with known inhibitory activity, or non-selective compounds, were not considered. Some such compounds include quinazoline and 1,2,3,4-dithiadiazole derivatives.

### 5.1. Quinazoline Derivative EVP4593

The quinazoline scaffold (also known as benzopyrimidine), along with piperidine and imidazole, is one of the most common and biologically significant structures in medicinal chemistry, possessing a unique combination of properties that make it a valuable pharmacophore. It is an aromatic heterocycle with a bicyclic structure consisting of two fused six-membered aromatic rings, a benzene ring and a pyrimidine ring [105]. Key advantages of the quinazoline scaffold include acceptable metabolic stability compared to some other heterocyclic structures, a wide range of lipophilicity and solubility, and the ability to form hydrogen bonds, which is necessary to ensure strong interactions at the binding site of the target protein.

EVP4593 is a quinazoline derivative substituted by [2-(4-phenoxyphenyl)ethyl]amino and amino groups at positions 4 and 6, respectively (Table 1). This compound is widely known as an inhibitor of the NF-*κ*B signaling pathway (IC_50_ 11 nM) [106]. At high concentrations it can cause apoptosis (cell death) by completely suppressing mitochondrial respiration, which is a potentially severe side effect. Additionally, its direct molecular target is not fully understood, and there are potential confounding effects from its inhibition of both SOCE and the downstream NF-*κ*B signaling pathway [106]. In human neuroblastoma SK-N-SH cells expressing full-length mutant huntingtin, EVP4593 at a concentration of 300 nM was shown to reversibly block pathologically elevated SOCE, demonstrated by whole-cell patch-clamp recordings and Fura-2/AM-based calcium imaging [107]. Similar results were observed in neurons differentiated from HD-specific embryonic stem cells, where Orai- and TRPC-containing SOC channels showed sensitivity to treatment with 100 nM EVP4593 measured using the same patch-clamp technique [108]. Among the proposed mechanisms modulating SOCE, the literature also cites an effect on mitochondrial complex I [109,110]. In silico docking simulations demonstrate that EVP4593 can bind directly to mitochondrial complex I. Long-term exposure to EVP4593 results in a decrease of huntingtin and STIM2 expression in GABA-ergic medium spiny neurons differentiated from HD-specific induced pluripotent stem cells that was analyzed by Western blotting of protein lysates from treated neurons [61,62,111]. A comprehensive review of EVP4593′s effects was previously published by our research group [63].

### 5.2. 1,2,3,4-Dithiadiazole Derivatives

The 1,2,3,4-dithiadiazoles have recently been investigated as potential modulators of calcium signaling [63,112,113]. The structure of these compounds is based on a five-membered heterocyclic moiety containing sulfur and nitrogen atoms. The thiadiazole ring is thought to act as a hydrogen-binding system and as a two-electron donor [114,115,116]. Five-membered heterocyclic compounds exhibit diverse biological activity, likely due to the presence of the –N=C–S– group. Furthermore, thiadiazoles readily penetrate cell membranes due to their mesoionic nature, and their high lipid solubility is explained by the presence of a sulfur atom.

In a series of studies, Grekhnev et al. [63,112,113] identified 3-(4-nitrophenyl)-5-phenyl-3H-1,2,3,4-dithiadiazole-2-oxide (compound 6) as a promising SOCE inhibitor with an IC_50_ of 6.2 μM for inhibition of Tg-induced calcium response in HEK293 cells measured by fluorescence calcium imaging with Fluo-4AM or Fura-2AM (Table 1) [63]. Another promising derivative was (3-(3,5-difluorophenyl)-5-phenyl-3H-1,2,3,4-dithiadiazole-2-oxide) (compound 2), which exhibited an IC_50_ of 7.4 μM [112]. SAR analysis revealed that electron-withdrawing substituents, such as the nitro group or difluorophenyl, may enhance the inhibitory activity. This was concluded from comparative dose–response curves (IC_50_ values) obtained by calcium imaging. These compounds are hypothesized to directly inhibit SOC channels activated by both STIM1 and STIM2, confirmed by whole-cell patch-clamp recordings and experiments on HEK293 lines with STIM1/STIM2 knockout or overexpression. Furthermore, impaired calcium signaling may be partially due to modulation of the physicochemical properties of ER membranes or direct effects on proteins involved in calcium release, such as inositol triphosphate receptors [117]. Further studies will be focused on introducing substituents into the second benzene ring to improve the activity and selectivity of these compounds.

### 5.3. 3,5-Bis(benzylidene)-4-Piperidone Derivatives

The 3,5-bis(benzylidene)-4-piperidone (BAP) is a synthetic heterocyclic analog of the natural polyphenol curcumin (1,7-bis(4-hydroxy-3-methoxyphenyl)-1,6-heptadiene-3,5-dione). Unlike curcumin, which contains an open seven-carbon chain with two α,β-unsaturated ketone groups, BAP features a central chain replaced by a rigid six-membered piperidine ring (piperidin-4-one). Exocyclic benzylidene groups are located at the 3- and 5-positions of this ring, forming two α,β-unsaturated double bonds.

The central mechanism of action involves the ability of 3,5-BAPs to act as classical Michael acceptors. Due to the presence of two α,β-unsaturated carbonyl groups (the exocyclic double bonds at the 3- and 5-positions), these compounds exhibit high electrophilicity. The intracellular nucleophile with which they react is the thiol group of a critical cysteine residue (Cys38) located in the DNA-binding domain of the NF-*κ*B p65 subunit [118].

Since NF-*κ*B is a transcription factor that controls the expression of numerous genes, including those responsible for the formation of SOCE channels, inhibition of NF-*κ*B leads to reduced transcription of Orai1 and STIM1 [119]. Thus, 3,5-BAPs and their conjugates are promising compounds for cancer therapy, acting through the indirect regulation of SOCE, disrupting calcium homeostasis, and leading to apoptosis. Moreover, because SOCE is well known to be the initial step required for activation of the NF-κB signaling pathway, inhibitors of SOCE are often found to act as negative modulators of NF-κB. Such direct modulation of SOC channels has been demonstrated for EVP4593 [63,107] and remains to be examined for 3,5-BAPs.

## 6. Summary

Recent literature reviews have demonstrated significant progress in elucidating the molecular mechanisms of SOCE and in developing pharmacological tools to modulate this pathway. However, existing reviews, such as the work by Hala’ufia and Roman [119] and Liao et al. [120], primarily focus on the biological roles of Orai/STIM proteins in specific pathologies (e.g., triple-negative breast cancer and in cardiovascular diseases), offering only cursory mention of pharmacological tools without providing a detailed chemical analysis of the inhibitors. In contrast, the review by Mignen et al. [121] is confined to clinical candidates and covers a limited number of chemical classes, overlooking novel heterocyclic compounds, such as 1,2,3,4-dithiadiazoles, as well as systematic data on molecular interactions between inhibitors and key Orai channel residues. This gap in the literature underscores the need for a comprehensive review that integrates SAR data, binding mechanisms, and strategies for enhancing.

### 6.1. The Structure–Activity Relationships of SOCE Inhibitors

**Figure 1:**
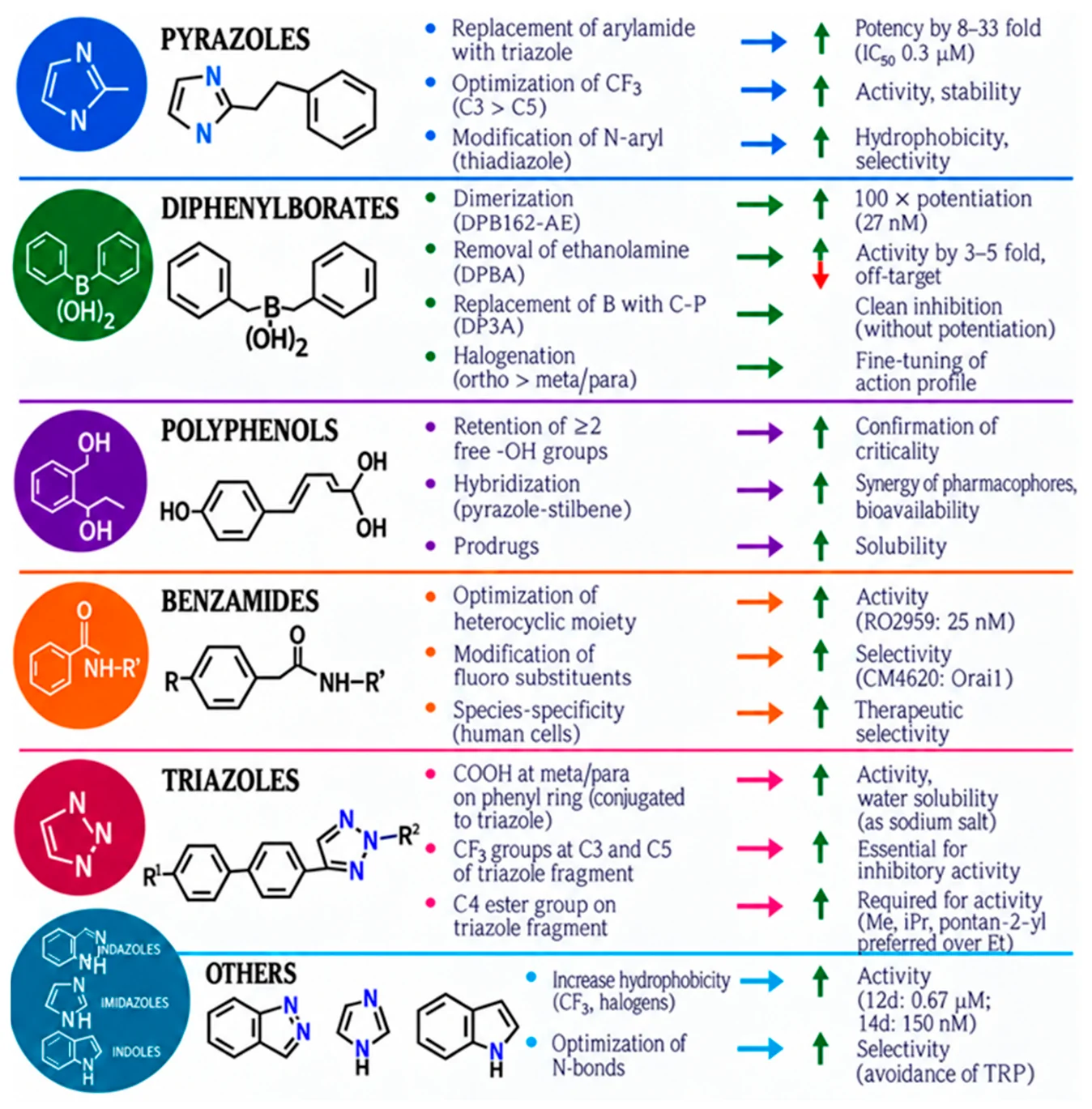
Possible strategies for the rational design of SOCE inhibitors. green upward-pointing arrow—increase in efficiency, increase in effect; red downward-pointing arrow—decrease in efficiency.

Analysis of the SARs of various classes of Orai channel inhibitors reveals several functional determinants governing their inhibitory activity (Figure 1). Virtually all highly potent inhibitors are characterized by a rigid, predominantly planar hydrophobic core capable of interacting with the channel’s transmembrane domains. In the case of pyrazoles (such as BTP2 and GSK-7975A), the presence of two aryl substituents on the pyrazole ring creating a rigid, hydrophobic structure is crucial [40,41]. Replacing these substituents results in a loss of activity. Similarly, the conformational rigidity of the stilbene moiety plays a critical role in polyphenols (such as resveratrol and diethylstilbestrol): *trans*-stilbene, which lacks hydroxyl groups, is inactive [79]. This confirms the necessity of both hydrophobicity and a fixed, rigid conformation to ensure proper orientation within the binding site. The orientation of the amide linker can also influence the structural rigidity of inhibitors. For classes of compounds containing an amide group (such as indazoles and benzamides), linker orientation is a critical factor determining activity [48,95]. In the series of indazole-3-carboxamides, it was demonstrated that a “forward” linker (-CO-NH-Ar) yields significantly higher activity (IC_50_ = 0.67–1.51 µM) than a “reverse” linker (-NH-CO-Ar), for which the IC_50_ exceeds 29 µM [48]. This indicates strict steric and electronic requirements regarding the geometry of the interaction with the binding site.

Furthermore, the presence of polar groups, such as hydroxyl (-OH) or amide (-CONH-) moieties, can be critical for the inhibitory potency of the compounds, as these groups can participate in hydrogen bonding with amino acid residues within the active site of the Orai channel. For instance, in the case of polyphenols (resveratrol and genistein), the presence of hydroxyl groups is crucial for their activity [79]. This highlights the need for a balance between the lipophilic and hydrophilic properties of the molecule to ensure optimal interaction with Orai.

Across all compound classes, specific substituents that mediate polar interactions (hydrogen bonds) or modulate the electron density of aromatic rings are essential for effective inhibition, determining the binding affinity and selectivity. In particular, in the pyrazole class, the CF_3_ group at the C3 position is necessary for creating steric and lipophilic properties, as well as participating in hydrophobic interactions with Val106 and Val198 [38,43]. Replacing CF_3_ with a methyl group reduces or abolishes activity [38]. Significantly, the position of CF_3_ is critical, as indicated by the decreased activity of derivatives with CF_3_ at C5.

The introduction of halogens, particularly in diphenylborates (halogenated analogs of 2-APB) and benzamides (Synta66 and CM4620), significantly influences activity depending on the size and electronegativity of the halogen, and also enhances hydrophobic contacts and *π*-*π* stacking. Thus, the inhibitory activity of *meta*- and *para*-halogenated derivatives increases with increasing atomic size and decreasing electronegativity (*m*-F < *m*-Cl < *m*-Br < *m*-I) [75,95]. Furthermore, the position of the halogen critically influences the mode of action: *ortho*-substituted 2-APB analogs enhance SOCE, while *meta*- and *para*-substituted derivatives inhibit it.

### 6.2. Selectivity of SOCE Inhibitors

Currently, the highest proven selectivity for Orai channels has been established for several classes of small-molecule inhibitors, with pyrazoles, benzamides, indazole-3-carboxamides, and polyphenols playing key roles. Within the pyrazole class, the most promising compound is GSK-7975A, which inhibits SOCE with an IC_50_ of approximately 4 µM in HEK293 cells and ~10 µM in Jurkat T cells. Its key advantage is high selectivity over voltage-gated calcium channels, as well as direct interaction with the Orai1 selectivity filter, a fact confirmed by mutational analysis. Among benzamides, a promising compound is CM4620 (IC_50_ ≈ 0.1 µM in vascular smooth muscle cells)—it not only exhibits the lowest inhibitory concentration known for this class but is also undergoing clinical trials. In the indazole-3-carboxamide class, compound 4k (IC_50_ ≈ 4.9 µM) is of particular value—it completely blocks CRAC currents and does not affect other channels at concentrations up to 30 µM. This makes it a highly suitable tool for the selective targeting of Orai1. Among the polyphenols, the most promising compound is AnCoA4—developed from biochanin A and genistein. At a concentration of 20 µM, it reduces calcium currents by 80% through direct binding to the C-terminal region of Orai1, with an excess of STIM1 virtually abolishing its inhibitory effect, indicating a competitive interaction.

In contrast, despite their historical significance, 2-APB and SKF-96365 are now considered to have limited utility due to their multi-target profiles and biphasic effects, which significantly complicate the interpretation of experimental data.

The current range of selective STIM inhibitors consists primarily of indole-3-carbinol derivatives (femindol) and aminoglycosides (G418). Further development is needed to establish novel compounds capable of discriminating between SOC channels activated by STIM1 and STIM2.

Thus, the modern arsenal of selective SOCE modulators relies predominantly on pyrazoles, benzamides, indazole-3-carboxamides, and polyphenols for Orai, as well as on compounds that specifically disrupt the STIM–Orai protein–protein interaction to target STIM.

### 6.3. Mode of Action of SOCE Inhibitors

**Figure 2:**
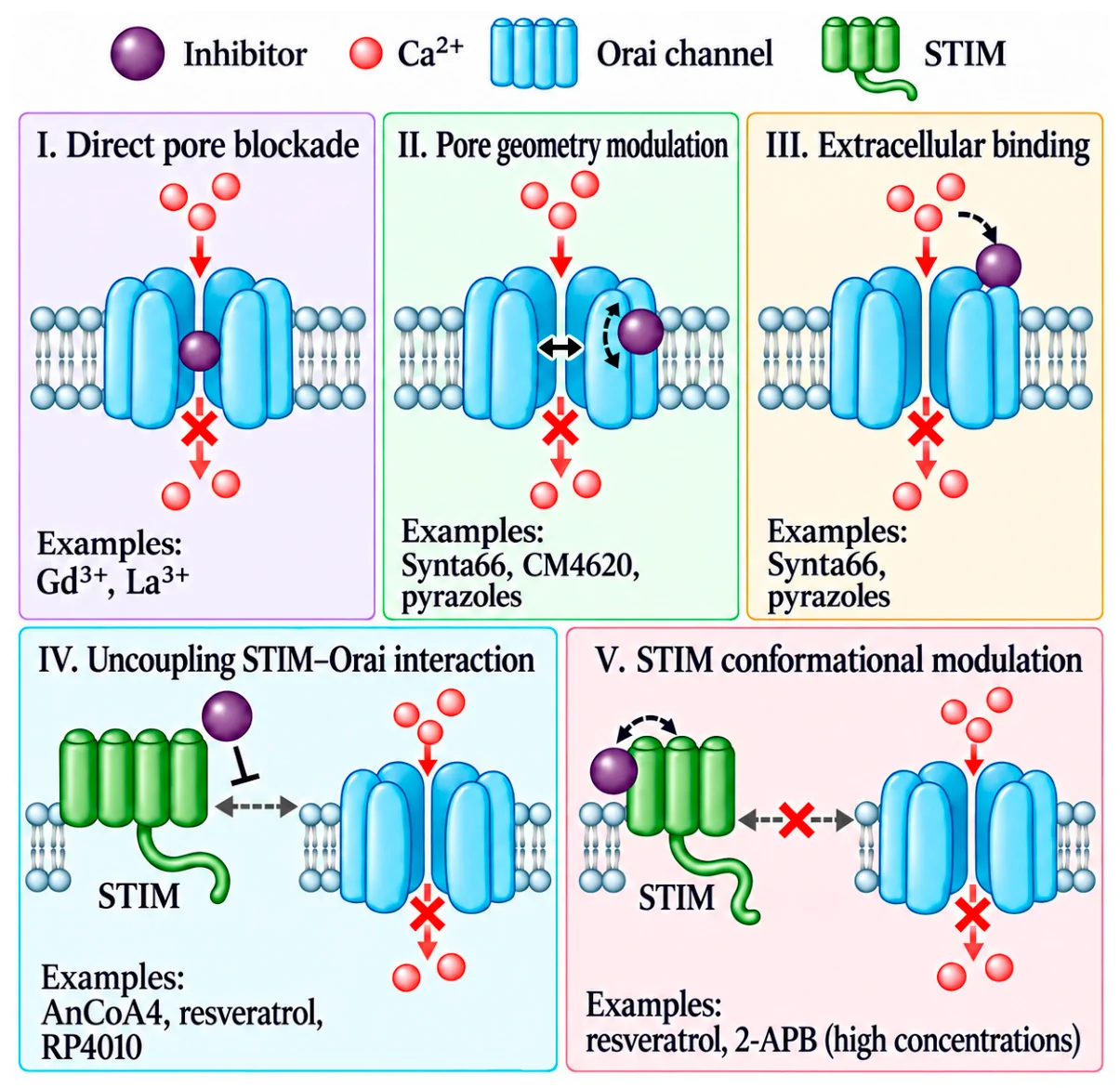
The principal mechanisms of action of SOCE inhibitors. The Orai channel and the STIM proteins are shown schematically.

Analysis of the available data reveals a number of general principles and patterns underlying the pharmacology of Orai and STIM protein inhibitors. Despite their significant structural diversity, these inhibitors share characteristic features in their interaction with the molecular target. A summary diagram of the modes of interaction between SOCE inhibitors and the Orai channel and STIM proteins is presented in Figure 2.

The modes of interaction between inhibitors and Orai channels are not limited to simple physical occlusion of the pore. Virtually all the compounds affect the conformational dynamic of proteins, either by stabilizing the closed state (pyrazoles and polyphenols) or, conversely, by disrupting the physiological interaction between STIM1 and Orai1 (2-APB, AnCoA4, and benzamide derivatives compound 36) [47,55,95]. Another possibility is that a biphasic mode of action is an inherent characteristic of certain ligands. In particular, 2-APB potentiates the CRAC activity at low concentrations while inhibiting it at high concentrations. In the case of pyrazole Pyr6, the extracellular site of the compound merely initiates the process, while membrane penetration provides access to intracellular binding sites [43].

A key feature of most compounds is their ability to act on the pore region of the channel and modify the selective filter and hydrophobic gate, which leads to a change in the channel geometry and disruption of ionic conductivity. This mechanism has been studied in detail using pyrazoles, diphenylborates, and indole-containing compounds and supported by molecular modeling and functional experiments [43,77,88]. The data indicate that pyrazoles interact with the extracellular surface of the channel in the region of the M1 helices of two adjacent subunits in the selective filter region, forming bonds with residues Val106 and Val198 and hydrogen bonds with Gln109 [43]. Indoles can bind directly to the selective filter region and the outer region of the pore [89,90]. Diphenylborate also has a direct effect on the pore. Molecular docking revealed that 2-APB is anchored in the selective filter region, where its amino group electrostatically binds to the carboxyl groups of Glu106 of adjacent subunits, while the aromatic rings form *π*-*π* stacking with Phe99 and hydrophobic contacts with Val102, stabilizing the channel in a closed conformation [77]. A key consequence of this interaction is a direct change in pore geometry. This is further supported by the fact that mutations that widen the pore (e.g., V102A/C/G) or disrupt selectivity (E106D) dramatically reduce the blocking activity of these inhibitors. Similarly, polyphenols (resveratrol and diethylstilbestrol) have been shown to compete with calcium for a common binding site, indicating interaction with an allosteric site within the pore region [79].

Another potential mechanism involves targeting the functional interaction between the STIM sensor and the Orai pore-forming subunit. Here, the target is not the ion channel itself but rather the dynamic protein complex that mediates signal transmission from the ER to the plasma membrane. One possible mode of action is the functional uncoupling of STIM1 and Orai, characterized by the inhibition of activating signal transmission while the physical association between the proteins remains intact. This mechanism has been studied in detail using diphenylborates [55]. Literature findings have shown that these compounds can disrupt the functional coupling between STIM and Orai proteins, thereby inhibiting calcium channel activation. A key element of this process is a specific amino acid residue in position 85 in the ETON domain within the Orai1 that transmits an inhibitory signal without affecting the physical interaction between the proteins [55].

An alternative mechanism influencing the interaction between STIM and Orai involves direct competition by a ligand for the STIM-binding site on the Orai C-terminal domain. The most prominent example of this group is the flavonoid AnCoA4. Molecular modeling has shown that AnCoA4 binds to the Orai1 C-terminal domain, competing with STIM1 for a binding site that includes residues His299, Glu318, and Phe300. This binding reduces the efficiency of STIM1 recruitment to the channel, resulting in the inhibition of SOCE [47]. Unlike 2-APB, this mechanism supposes the physical displacement of STIM1 rather than functional uncoupling.

RP4010 serves as an intriguing example of an inhibitor operating via this mechanism. This compound is an experimental drug developed by Rhizen Pharmaceuticals and is being investigated as an antitumor agent. Its precise structural formula and chemical name are protected by the developer’s commercial patent and are rarely explicitly published in public databases. The compound is believed to target the membrane structure of the Orai1/STIM complex, disrupting the coupling between STIM1 and Orai1 [122].

Finally, a number of inhibitors, including certain polyphenols and steroids (e.g., 17*β*-estradiol), act at an earlier stage by disrupting the STIM1 oligomerization process. Since the formation of STIM1 multimeric complexes is a prerequisite for its subsequent interaction with Orai, blocking this step effectively inhibits CRAC channel activation [56,57,102]. This mechanism differs from previous approaches, which target the sensor itself rather than the STIM1–Orai complex, making this approach more universal, as it abolishes the activation of all types of Orai channels.

## 7. Conclusions

In recent years, significant efforts have been directed toward developing effective SOCE inhibitors based on diverse pharmacophores. Despite considerable progress in this area, the application of promising compounds remains constrained by substantial limitations. In particular, pyrazole derivatives, such as BTP2 and GSK-7975A, are potent CRAC channel inhibitors but suffer from nonspecific activity and unclear mechanisms of action. Diphenylborate derivatives, including 2-APB, are widely used to characterize CRAC channel activity; nevertheless, their bimodal effects and lack of selectivity complicate data interpretation. Polyphenols such as trans-resveratrol and DES exhibit inhibitory activity against calcium signaling, but the structural determinants conditioning their activity remain poorly defined. Indazole derivatives, including 3-aminoindazoles and indazole-3-carboxamides, demonstrate calcium influx inhibitory activity; however, their high hydrophobicity limits their practical utility. Imidazole derivatives, such as SKF-96365, were among the first identified CRAC channel inhibitors but are hindered by nonspecific activity. Consequently, further elucidation of the mechanisms of action of these compounds and the development of more selective and potent inhibitors are necessary. The application of structure-based drug design and high-throughput screening may facilitate the discovery of novel compounds with improved pharmacological profiles. In general, for next-generation SOCE inhibitors, the critical requirements are as follows: (1) high selectivity for the target (Orai1 versus Orai2/Orai3, as well as a lack of effect on TRP channels, thereby avoiding unwanted side effects and ensuring the purity of the pharmacological signal), (2) a confirmed binding mechanism—verified by both in silico and in vitro approaches, providing a rational basis for further optimization, and (3) pharmacological properties suitable for clinical practice—an optimal balance of lipophilicity (clogP ≤ 3.60), water solubility, bioavailability, predictable metabolism, and an absence of toxicity upon long-term use, all of which are prerequisites for the transition from experimental tools to therapeutic agents. Nevertheless, SOC channels remain attractive targets for drug development, as evidenced by the advancement of SOCE inhibitors into clinical trials.

## Figures and Tables

**Table 1:** Chemical structure and inhibitory concentration of SOCE.

Inhibitors of SOCE	Chemical Structure	Inhibitory Concentration of SOCE	Target Experimental Cell Lines
*Orai-associated small-molecule inhibitors*			
Pyrazole Derivatives			
GSK-5498A (2,6-difluoro-N-[1-[[2-(2-methyl-5-thienyl)phenyl]methyl]piperidin-4-yl]benzamide)	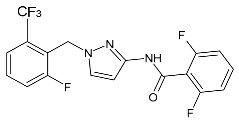	0.22–3.1 μM	Jurkat T cells [35]
			HEK293 cells [36,37,38,39]
GSK-5503A (2,6-difluoro-N-(1-(2-phenoxybenzyl)-1H-pyrazol-3-yl)benzamide)	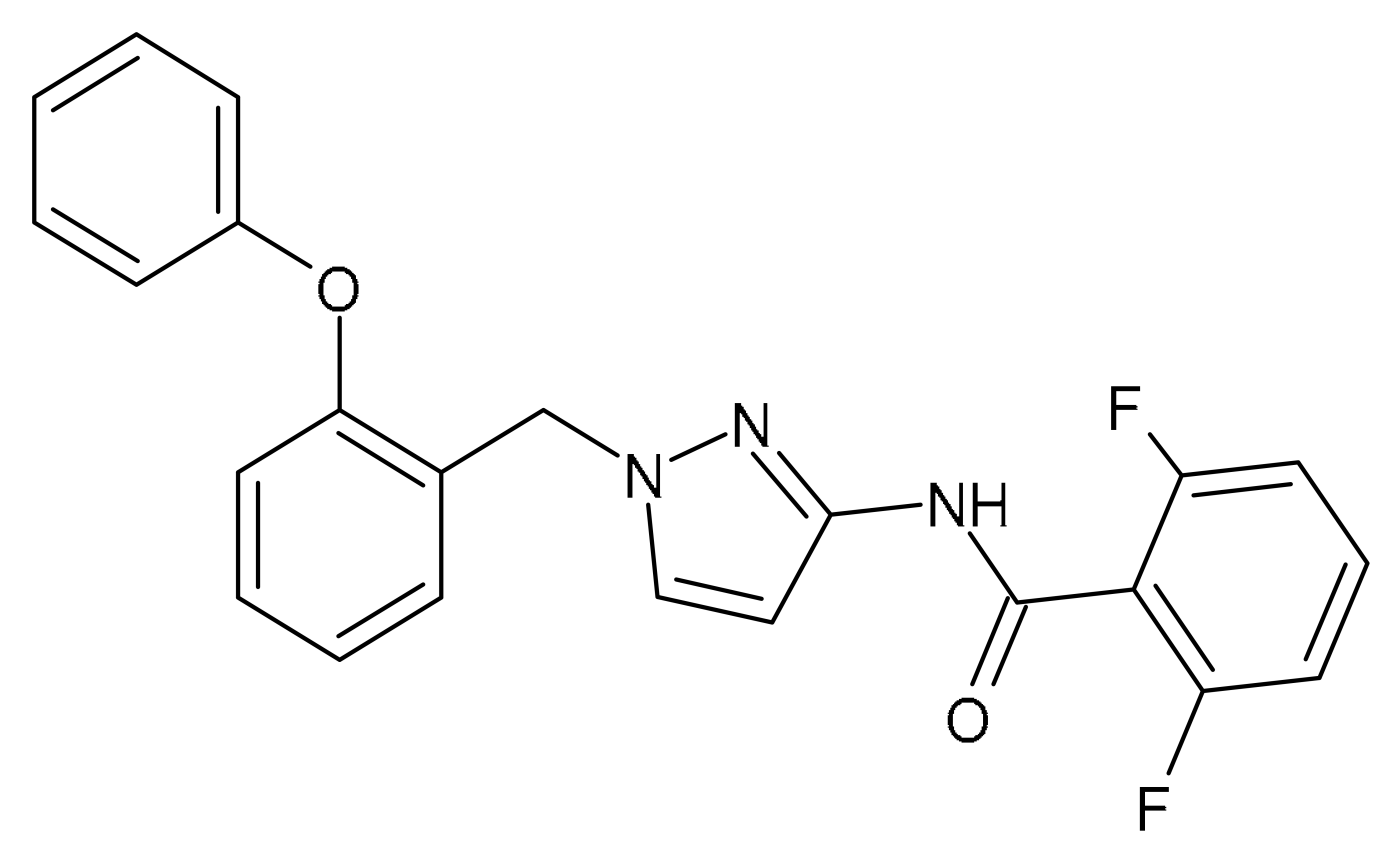	~4 μM	HEK293 cells [37,38,40]
GSK-7975A (2,6-difluoro-N-(1-(4-hydroxy-2-(trifluoromethyl)benzyl)-1H-pyrazol-3-yl)benzamide)	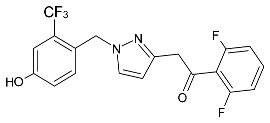	~4 μM	HEK293 cells [37,40]
		~10 μM	Jurkat T cells [36,41]
BTP2 (N-[4-[5-bis(trifluoromethyl)-1H-pyrazol-1-yl]phenyl]-4-methyl-1,2,3-thiadiazole-5-carboxamide)	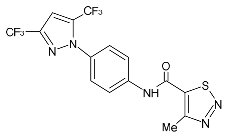	10–100 nM	Jurkat T cells [34,42]
			Jurkat T cells [33,43]
Diphenylborate Derivatives			
2-APB (2-(diphenylboranyloxy)ethan-1-amine)	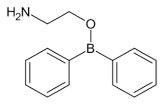	1–50 μM	Jurkat T cells [44]
		3–50 μM	HEK293 cells [45]
Polyphenols			
resveratrol	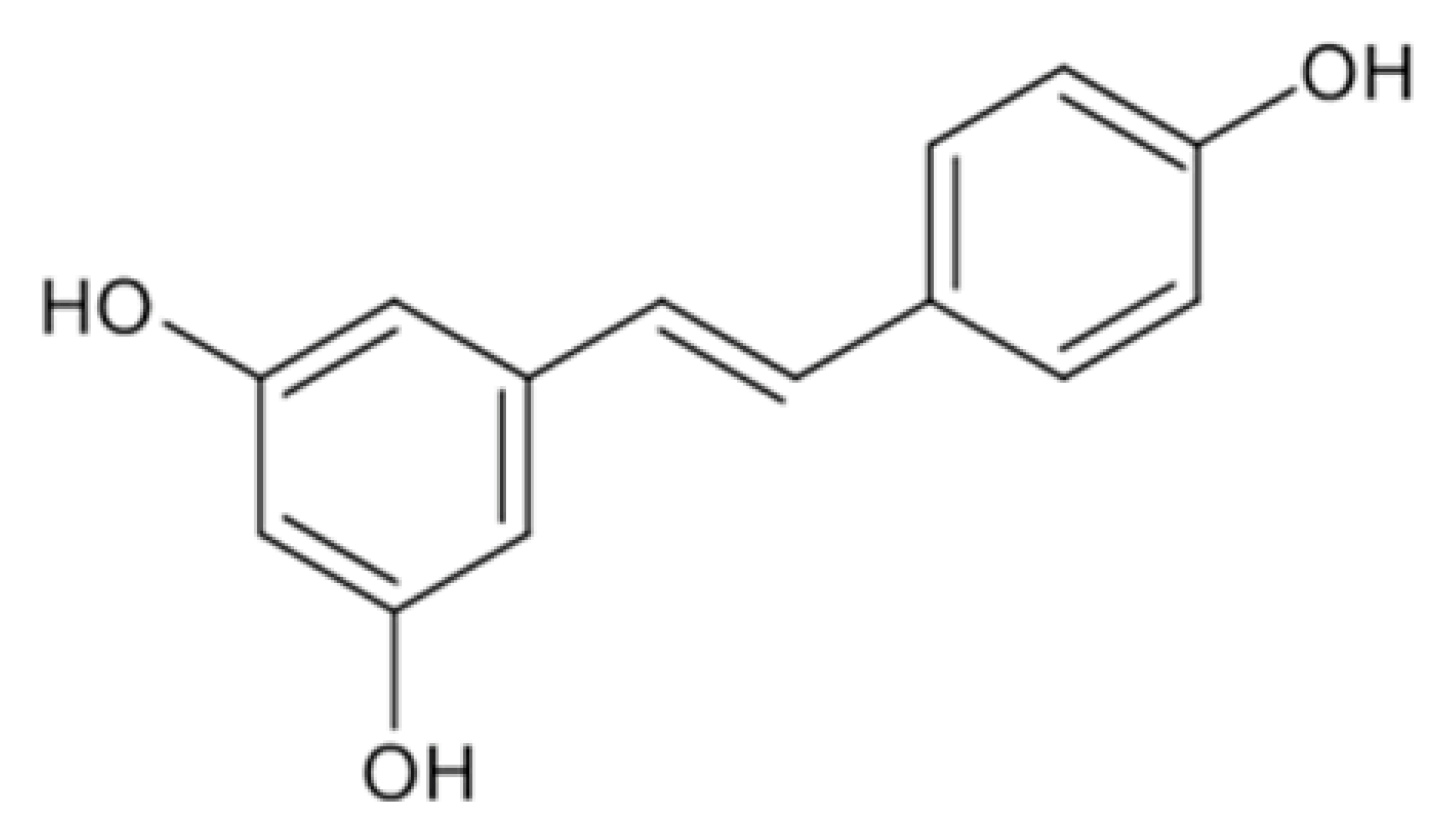	0.5 µM	thrombin-stimulated platelets [46]
AnCOA4 (3-(6-methoxy-1,3-benzodioxol-5-yl)-8,8-dimethyl-4H,8H-pyrano [3-f]chromen-4-one)	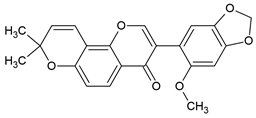	20 µM AnCoA4 resulted in 80%SOCE inhibition in HEK293 cells	HEK293 cells [47]
Indazole Derivatives			
The indazole-3-carboxamide series, compound (12 d)	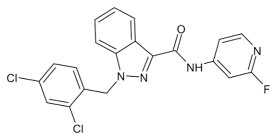	0.67 µM	RBL-2H3 cells [48]
Imidazole Derivatives			
SKF-96365 (1-(2-(4-methoxyphenyl)-2-(3-(4-methoxyphenyl)propoxy)ethyl)-1H-imidazole hydrochloride)	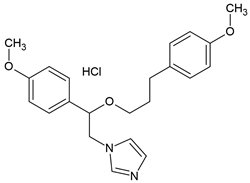	12 µM	Jurkat T cells [49]
Indole Derivatives			
The 7-azaindole series, compound (14 d)	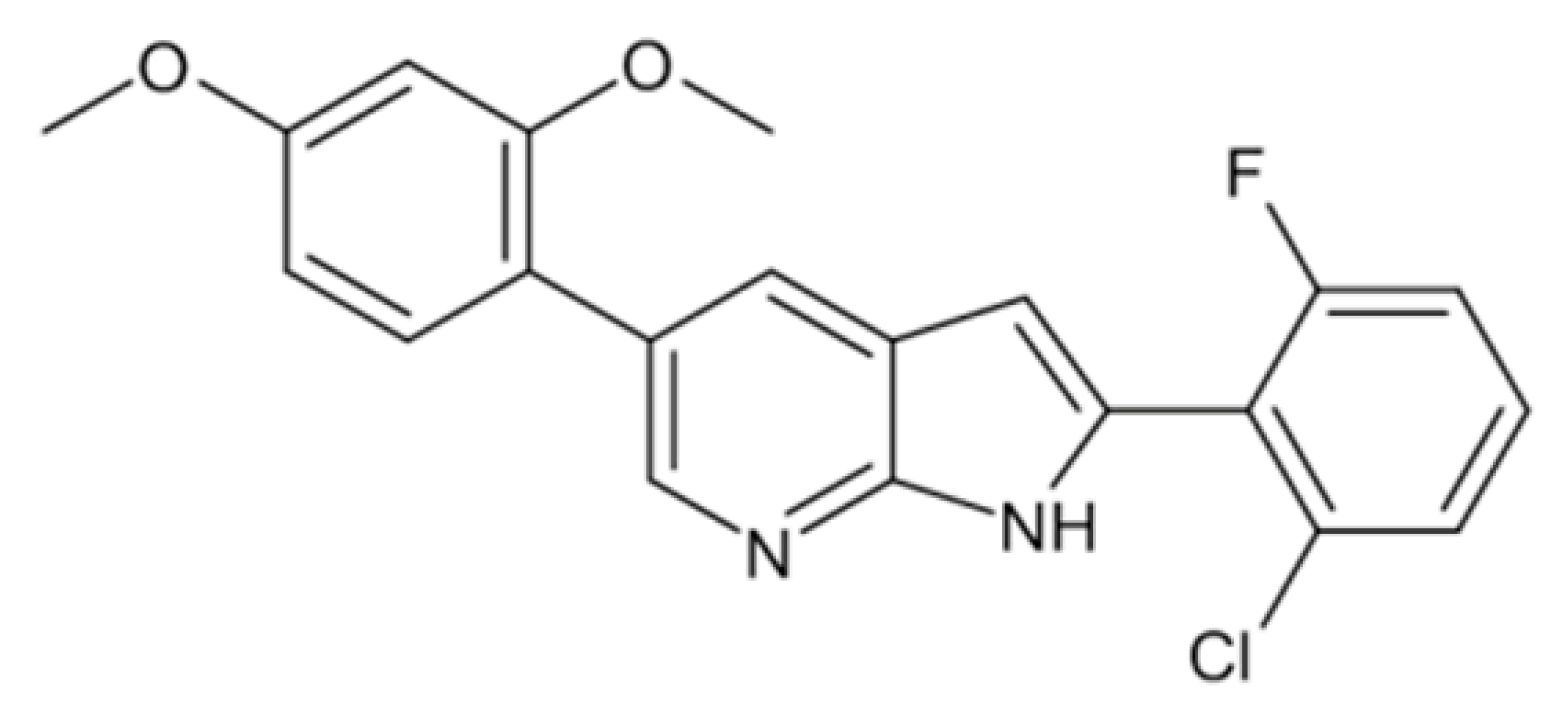	150 ± 22 nM	Jurkat T cells [50]
Benzamide Derivatives			
Synta66 (N-(2′,5′-dimethoxy [1′-biphenyl]-4-yl)-3-fluoro-4-pyridinecarboxamide)	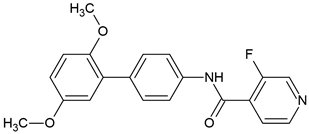	3 µM	RBL cells [51]
		26 nM	vascular smooth muscle cells [52]
CM4620 (N-(5-(6-chloro-2,2-difluorobenzo[d]dioxol-5-yl)pyrazin-2-yl)-2-fluoro-6-methylbenzamide)	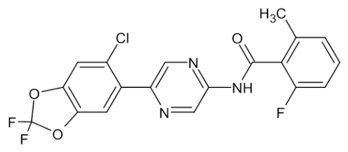	~0.1 µM	vascular smooth muscle cells [52]
Triazoles Derivatives			
CAI (carboxyamidotriazole)	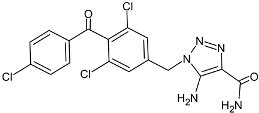	10 µM	CHO-K1 cells [53]
compound 34 (3-(1-(2′,3′-dimethoxy-[1,1′-biphenyl]-4-yl)-1H-1,2,3-triazol-4-yl)-benzoic acid)	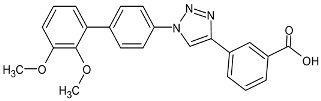	3 µM	HEK293 cells [54]
*STIM-associated small-molecule inhibitors*			
2-APB	––	50 µM	[55]
resveratrol	––	5–50 µM	[56]
17*β*-estradiol (steroid female sex hormone)	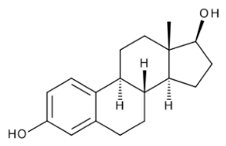	10 nM	HBECs [57]
femindol (indole-3-carbinol derivative)	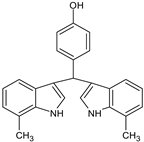	35 μM	MDAMB-231 cells [58]
4-MPTC (5-methyl-2-phenyl-2H-1,2,3-triazole-4-carboxylic acid)	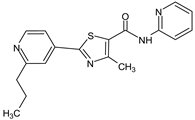	100 μM	HEK293 cells [59]
G418 (aminoglycoside antibiotic Geneticin)	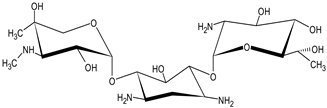	3–50 μM	HEK293 cells [60]
			HD76 neurons [61]
*Other compounds with inhibitory activity against SOCE*			
EVP4593 (4-N-[2-(4-phenoxyphenyl)ethyl]quinazoline-4,6-diamine)	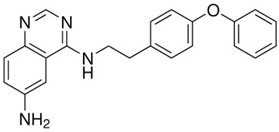	0.3 μM	MSNs Htt138Q-1exon [62]
compound 6 (3-(4-nitrophenyl)-5-phenyl-3H-1,2,3,4-dithiadiazole-2-oxide)	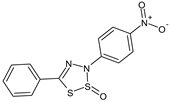	10 μM	HEK293 cells [63]

## Data Availability

No new data were created or analyzed in this study. Data sharing is not applicable to this article.

## Abbreviations

The following abbreviations are used in this manuscript:

FRET	Förster resonance energy transfer
*I_CRAC_*	Calcium current activated by calcium release
SOCE	Store-operated calcium entry
SAR	Structure–activity relationship
ER	Endoplasmic reticulum
IP_3_	Inositol-1,4,5-trisphosphate receptor
Tg	Thapsigargin
IC_50_	Half-maximal inhibitory concentration clinical trials
